# A heteromeric molecular complex regulates the migration of lung alveolar epithelial cells during wound healing

**DOI:** 10.1038/s41598-017-02204-2

**Published:** 2017-05-19

**Authors:** Manik C. Ghosh, Patrudu S. Makena, Joseph Kennedy, Bin Teng, Charlean Luellen, Scott E. Sinclair, Christopher M. Waters

**Affiliations:** 1 Department of Physiology, University of Tennessee Health Science Center, Memphis, TN 38163 USA; 2 Department of Medicine, University of Tennessee Health Science Center, Memphis, TN 38163 USA

## Abstract

Alveolar type II epithelial cells (ATII) are instrumental in early wound healing in response to lung injury, restoring epithelial integrity through spreading and migration. We previously reported in separate studies that focal adhesion kinase-1 (FAK) and the chemokine receptor CXCR4 promote epithelial repair mechanisms. However, potential interactions between these two pathways were not previously considered. In the present study, we found that wounding of rat ATII cells promoted increased association between FAK and CXCR4. In addition, protein phosphatase-5 (PP5) increased its association with this heteromeric complex, while apoptosis signal regulating kinase-1 (ASK1) dissociated from the complex. Cell migration following wounding was decreased when PP5 expression was decreased using shRNA, but migration was increased in ATII cells isolated from ASK1 knockout mice. Interactions between FAK and CXCR4 were increased upon depletion of ASK1 using shRNA in MLE-12 cells, but unaffected when PP5 was depleted. Furthermore, we found that wounded rat ATII cells exhibited decreased ASK1 phosphorylation at Serine-966, decreased serine phosphorylation of FAK, and decreased association of phosphorylated ASK1 with FAK. These changes in phosphorylation were dependent upon expression of PP5. These results demonstrate a unique molecular complex comprising CXCR4, FAK, ASK1, and PP5 in ATII cells during wound healing.

## Introduction

Epithelial repair mechanisms are initiated immediately following lung injury and involve an acute inflammatory response, immune cell recruitment, and activation of the coagulation cascade (reviewed in ref. 1). Nearby facultative progenitor cells, primarily alveolar type II cells (ATII) in the alveolus, rapidly migrate and spread to cover the denuded surface, while circulating stem cells and other progenitor cells are later recruited to the site of injury^2–6^. Along with these recruited cells, ATII cells eventually proliferate and undergo phenotypic differentiation in order to re-establish the integrity and functional organization of the epithelial layer^7–12^. Thus, it is clear that epithelial repair is a dynamic process involving primarily cell spreading and cell migration in the early stages, and later involves recruitment, proliferation, and differentiation.

Focal adhesion kinase-1 (FAK), a non-receptor tyrosine kinase, has long been recognized as a key regulator of cell migration (reviewed in refs 13–15). We and others have previously shown that overexpression of FAK stimulates cell migration, while decreased expression or overexpression of negative regulators inhibits cell migration^16–20^. FAK regulates cell migration in response to a broad range of stimuli and through multiple signaling pathways, most prominently the Src family of kinases. Interactions with integrin receptors increases phosphorylation of FAK at Tyr^397^ which promotes binding of Src and the formation of complexes with other structural and signaling molecules^13, 21, 22^. For example, we previously found that cell migration in a scratch wound model was dependent upon FAK interactions with c-jun N-terminal kinase (JNK) mediated via JNK-interacting protein-3 (JIP3)^17^. Through these complexes, FAK promotes several elements of cell migration including membrane protrusion and focal adhesion turnover.

We recently demonstrated that wounded ATII cells secreted the chemokine CXCL12 which promoted cell migration and wound closure through binding to its receptor CXCR4^23^. CXCL12/CXCR4-induced cell migration was previously demonstrated in progenitor B cells to be dependent upon interactions between CXCR4 and FAK^24, 25^. However, although it was reported that CXCL12 stimulated the activation of FAK and its recruitment into lipid rafts, the molecular interactions between FAK and CXCR4 were not elucidated. In the current study we investigated the interactions between FAK and CXCR4 in migrating ATII cells following wounding. Also, since our previous studies identified FAK-mediated regulation of JNK in lung epithelial cell migration^17^, we hypothesized that apoptosis signal regulating kinase-1 (ASK1), which activates JNK, is part of the FAK complex that regulates cell migration. Knockdown or inhibition of ASK1 has been shown to either promote^26^ or diminish^27, 28^ cell migration in tumor cells, but this has not previously been investigated in ATII cells. Since these interactions may be dependent upon changes in phosphorylation of ASK1, we also investigated the role of protein phosphatase-5 (PP5), a key regulator of ASK1 activity^29, 30^. We identified a molecular complex of FAK, CXCR4, PP5, and ASK1 that changed in composition in cells following wounding and that was dependent upon changes in phosphorylation of both FAK and ASK1.

## Results

### FAK interactions are altered in ATII cells following wounding

**Figure 1:**
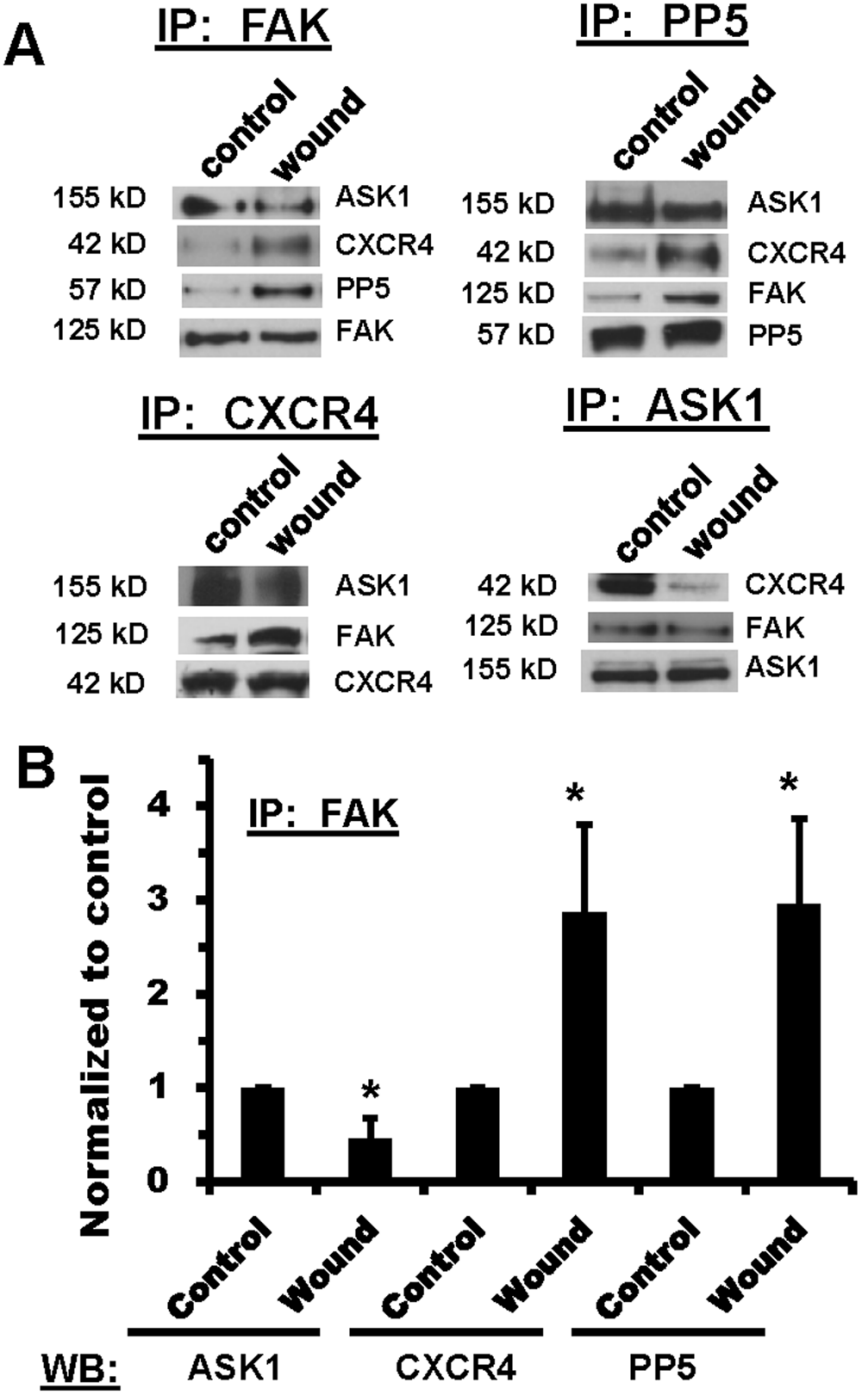
FAK interactions with CXCR4, ASK1, and PP5 were altered in wounded rat ATII cells. Cell lysates were collected from either unwounded monolayers or monolayers 24 hr after multiple scratch wounds to enrich for migrating cells. Equal amounts of protein were immunoprecipitated (IP) from cell lysates using antibodies against FAK, CXCR4, ASK1, and PP5, and then separated by gel electrophoresis followed by immunoblotting for the indicated proteins. Panel A Representative immunoblots showing interactions and the response to wounding. Each pair of bands shown was cropped from the same blot, and the full-length blots were provided in supplemental data. Each protein indicated was immunoblotted separately from independent gels. Panel B Densitometry analysis of results from FAK IP and immunoblot for ASK1, CXCR4, and PP5. Densitometry values were first normalized to FAK and then to the control unwounded condition. Asterisks (*) indicate a significant difference compared to control; (n = 4), p < 0.05.

To determine whether CXCR4 interacts with FAK in ATII cells, we performed immunoprecipitation (IP) studies in unwounded rat ATII cells and in cells 24 hr after multiple scratch wounds were applied to enrich the population of migrating cells. Figure 1A shows that CXCR4 interacted with FAK under control (unwounded) conditions, but the interaction increased in cells following wounding. These results were confirmed by immunoprecipitation with both a FAK antibody and a CXCR4 antibody followed by immunoblotting. Figure 1B provides quantitation for interactions using IP for FAK, indicating a significant increase in FAK/CXCR4 interactions in wounded cells. Similar to CXCR4, the interaction between PP5 and FAK was significantly increased in wounded cells. In contrast, the association between ASK1 and FAK was significantly decreased in wounded cells compared with control cells. These interactions were confirmed using IP with PP5 and ASK1 antibodies followed by immunoblotting. The results demonstrate basal interactions between FAK, CXCR4, ASK1, and PP5 that are enhanced in migrating cells for FAK, CXCR4, and PP5, but are diminished for ASK1.

### Wound healing is stimulated in cells deficient in ASK1, but inhibited in cells deficient in PP5

**Figure 2:**
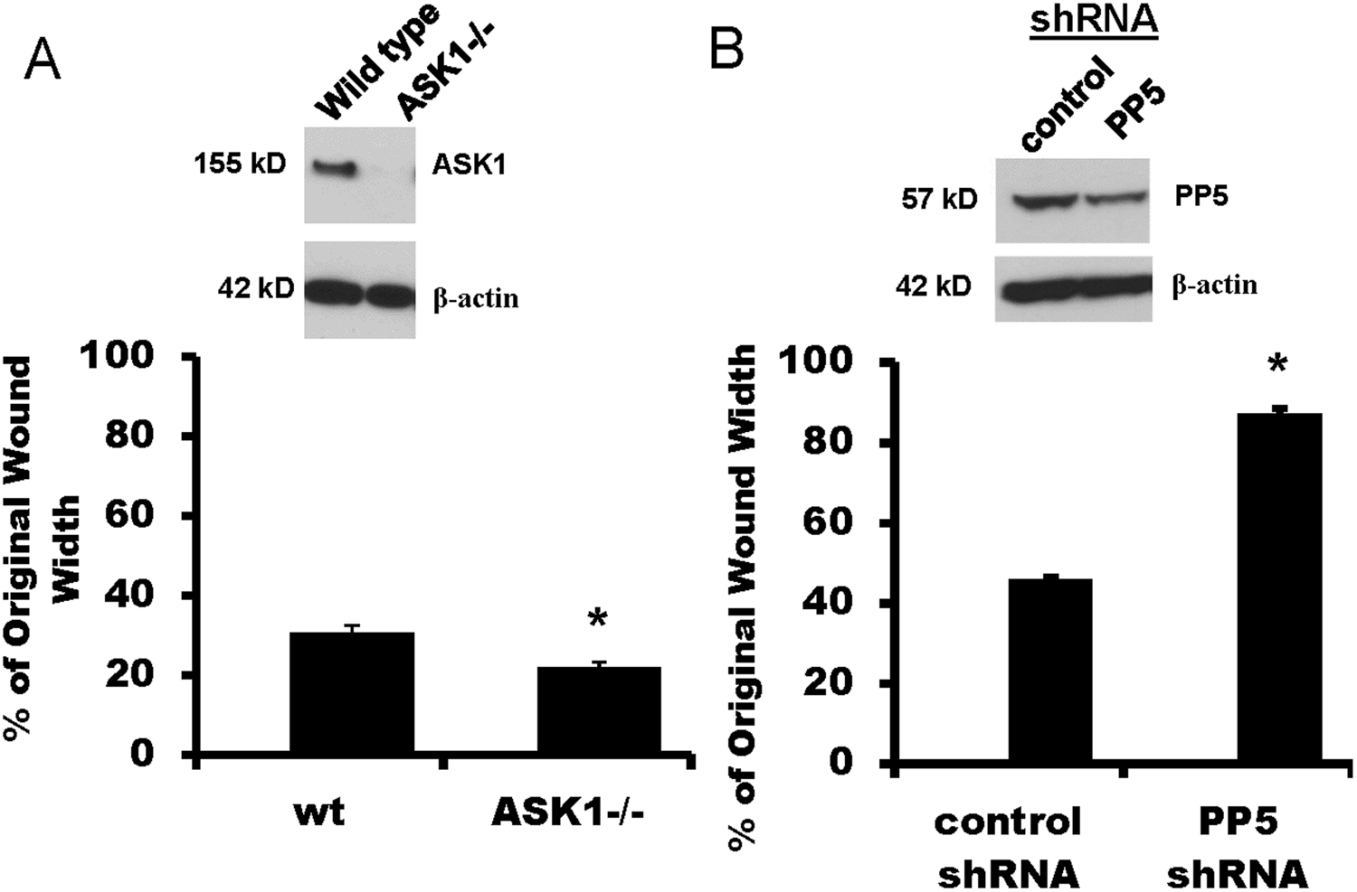
Wound healing was stimulated in ASK1-deficient cells, but inhibited in PP5-deficient cells. Scratch wounds were applied to confluent monolayers, and the wound widths were measured 24 hr later as a percentage of the original wound width. Panel A Primary ATII cells from ASK1−/− mice exhibited accelerated wound closure compared with ATII cells from wild type (wt) mice; wound closure measurements were made using the IncuCyte ZOOM system. Expression of ASK1 relative to β-actin is shown in the upper panel. Panel B Wound closure was inhibited in MLE-12 cells with PP5 expression knocked down by shRNA compared with control shRNA cells; wound closure was measured following multiple pipette tip scratch wounding. Expression of PP5 (relative to β-actin) was reduced by 40% (upper panel). Asterisks (*) indicate a significant difference compared to control; (n = 14 for Ask1−/− and n = 6 for PP5 shRNA), p < 0.05. Each pair of bands shown was cropped from the same blot, and the full-length blots were provided in supplemental data.

We previously showed that over-expression of FAK promoted epithelial wound closure while expression of a dominant negative form of FAK (FRNK) inhibited wound closure^17, 18^. We also showed that cells deficient in expression of CXCR4 exhibited decreased cell migration following wounding^23^. To determine whether decreased expression of ASK1 or PP5 would alter wound closure, we measured wound closure in a scratch wound assay in primary ATII cells from wild type and ASK1−/− mice and in MLE-12 cells expressing either control shRNA or PP5 shRNA. Figure 2 shows that ASK1 was not expressed in ATII cells from ASK1−/− mice and that PP5 expression was decreased in PP5-shRNA-MLE-12 cells (by approximately 40%, data not shown). Wound closure was significantly accelerated (smaller wounds at 24 hr) in ASK1−/− ATII cells compared with wild type ATII cells (Fig. 2A). MLE-12 cells deficient in expression of PP5 exhibited decreased wound closure (Fig. 2B).

### CXCR4-FAK interactions are inhibited by ASK1

**Figure 3:**
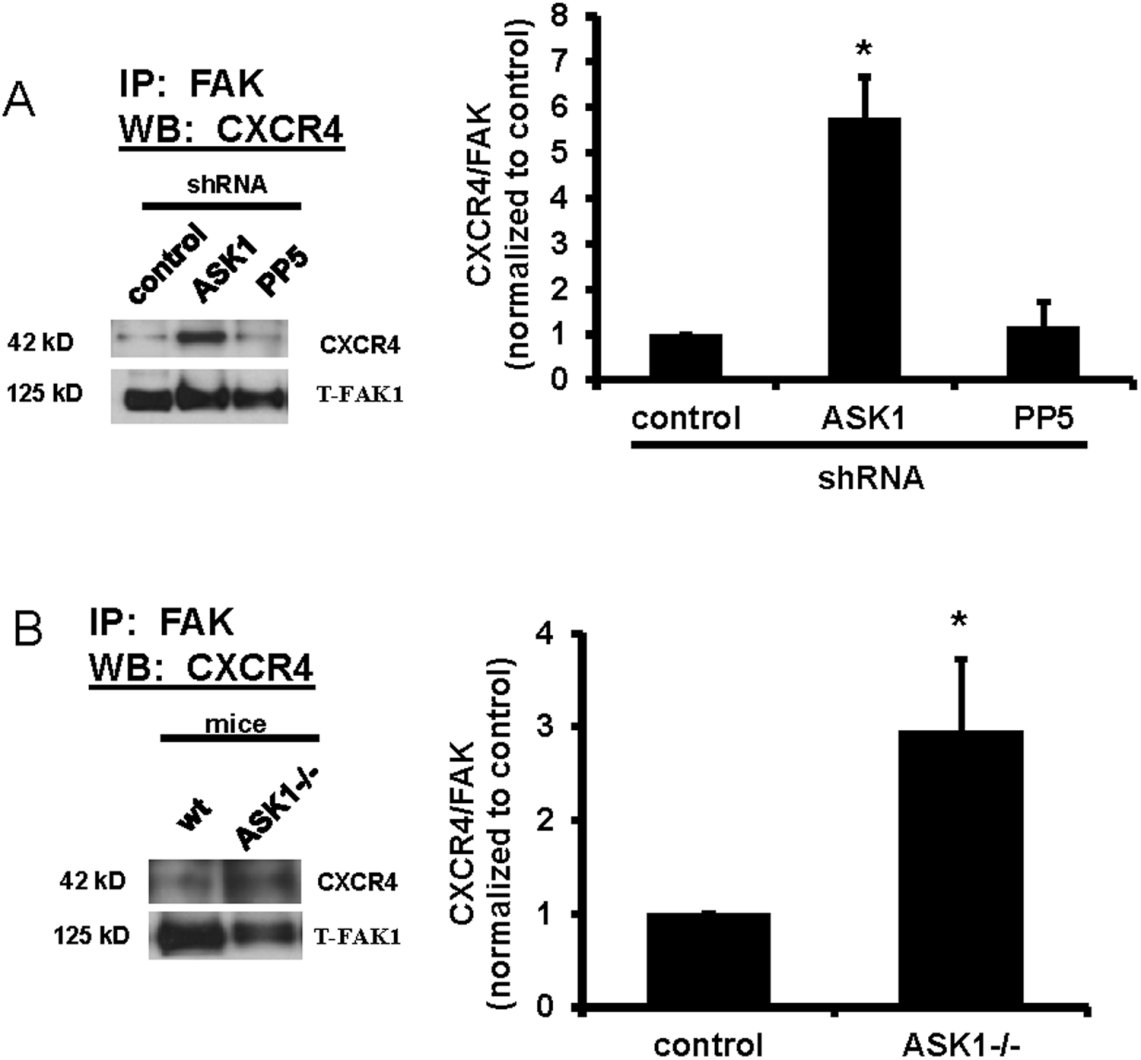
CXCR4 interactions with FAK were inhibited by ASK1. Panel A Cell lysates were collected from MLE-12 cells with control shRNA, ASK1-shRNA, or PP5-shRNA, immunoprecipitated with a FAK antibody, and immunoblotted for CXCR4 or FAK. Decreased expression of ASK1 caused a significant increase in CXCR4 associated with FAK as indicated by densitometry analysis (right panel), but decreased expression of PP5 did not affect the interaction (n = 3). Panel B CXCR4 interaction with FAK was significantly increased in cell lysates from ATII cells from ASK1−/− mice compared with cells from wild type (wt) mice (n = 5). Asterisks (*) indicate a significant difference compared to control (control-shRNA or wild type); p < 0.05. Total FAK was immunoblotted as a loading control. Each set of bands shown was cropped from the same blot, and the full-length blots were provided in supplemental data.

To determine whether the interactions between CXCR4 and FAK were affected by ASK1, IP experiments were performed using MLE-12 cells with shRNA knockdown of ASK1 (~65% reduction, data not shown) and with primary ATII cells from ASK1−/− mice. Figure 3 shows that CXCR4 immunoprecipitated with FAK was significantly increased when ASK1 expression was decreased by shRNA (Fig. 3A) or knockout (Fig. 3B). There was no effect on CXCR4/FAK interactions when PP5 expression was decreased by shRNA (Fig. 3A).

### Wounding promotes decreased serine phosphorylation of FAK and ASK1

**Figure 4:**
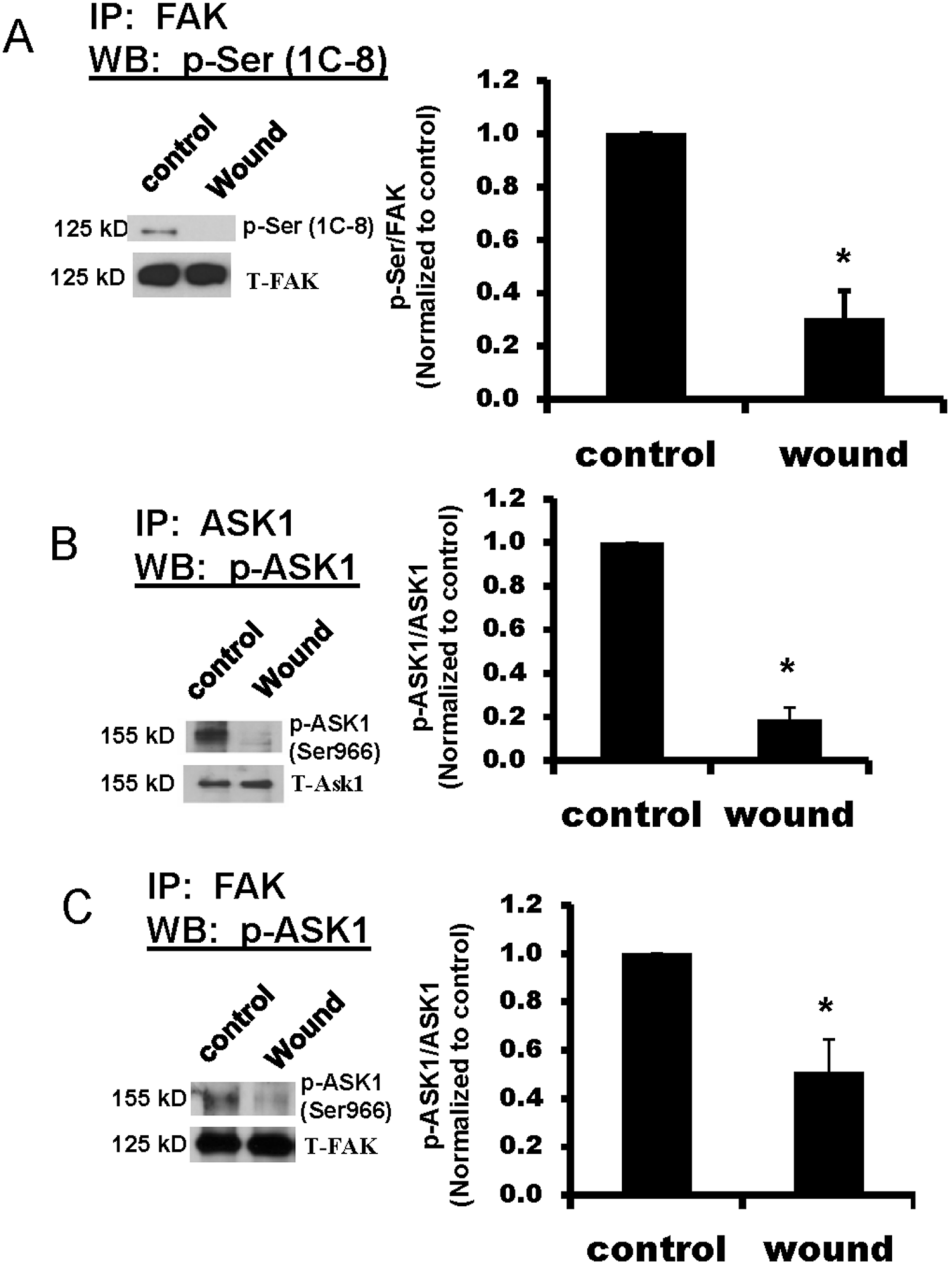
Serine phosphorylation of FAK and ASK1 were decreased in wounded cells. Cell lysates from unwounded and wounded primary rat ATII cells were collected after 24 hr, immunoprecipitated (IP) for FAK or ASK1, and then immunoblotted as indicated. Panel A Wounding caused a significant decrease in p-Ser-FAK recognized using the 1C8 antibody. Panel B Wounding caused a significant decrease in p-Ser^966^-ASK1. Panel C Wounding caused a significant decrease in p-Ser^966^-ASK1 associated with FAK. Left panels show representative blots, and right panels show densitometry with values normalized first to the loading control and then to the control unwounded condition. Asterisks (*) indicate a significant difference compared to control; (n = 4), p < 0.05. Each pair of bands shown was cropped from the same blot, and the full-length blots were provided in supplemental data. Each protein indicated was immunoblotted separately from independent gels.

We previously showed an increase in Tyr^397^ phosphorylation following wounding of lung epithelial cells^17^. However, the role of serine phosphorylation in the regulation of FAK and its interactions with other proteins is less well understood^31, 32^. To determine whether serine phosphorylation of FAK was altered in migrating cells, we used a FAK antibody for IP followed by western blotting for phospho-serine (p-Ser). When we used the monoclonal anti-phospho-serine antibody 1C8, we detected a significant decrease in FAK p-Ser in wounded cells compared with unwounded cells (Fig. 4A).

To determine whether ASK1 serine phosphorylation was altered in migrating rat ATII cells, we used an ASK1 antibody for IP and then immunoblotted using an antibody against p-Ser^966^-ASK1. Wounding caused a significant decrease in p-Ser^966^-ASK1 (Fig. 4B). Using a similar approach, we found no changes in p-Ser^83^-ASK1 following wounding (data not shown). Furthermore, when we used a FAK antibody for IP, there was a significant decrease in p-Ser^966^-ASK1 associated with FAK (Fig. 4C).

### Loss of serine phosphorylation of FAK and ASK1 in migrating cells is regulated by PP5

**Figure 5:**
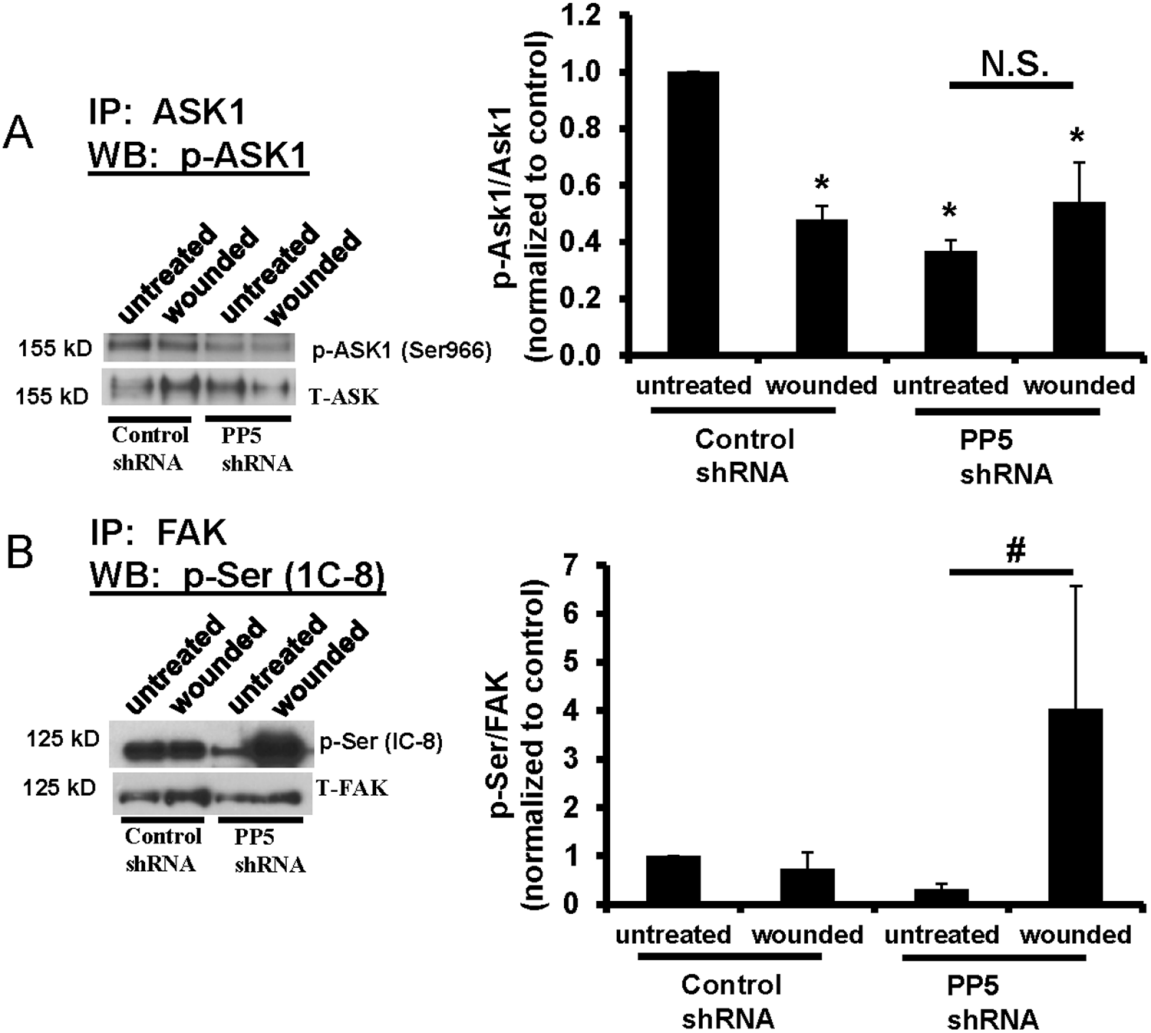
PP5 regulates the phosphorylation of FAK and ASK1 in wounded cells. Cell lysates from unwounded and wounded MLE-12 cells were collected after 24 hr, immunoprecipitated (IP) for FAK or ASK1, and then immunoblotted as indicated. Panel A Knockdown of PP5 using shRNA decreased p-Ser^966^-ASK1 in unwounded cells and showed no change in wounded cells. Panel B Knockdown of PP5 using shRNA caused a significant increase in p-Ser-FAK (recognized by the 1C8 antibody) in wounded cells. Right panels show the densitometry analysis normalized to unwounded controls. Asterisks (*) indicate a significant difference compared to control, (n = 4), p < 0.05. N.S. – no significant difference. Each set of bands shown was cropped from the same blot, and the full-length blots were provided in supplemental data. Each protein indicated was immunoblotted separately from independent gels.

Based upon our findings that there was an increase in PP5 association with FAK and a decrease in association with ASK1 in wounded cells (Fig. 1), we investigated whether decreased expression of PP5 affected serine phosphorylation. Figure 5A shows that p-Ser^966^-ASK1 was significantly decreased in unwounded MLE-12 cells with reduced PP5 expression, and there was no significant change in p-Ser^966^-ASK1 in wounded cells with PP5-shRNA. Although there was no significant difference in p-Ser-FAK in cells with control shRNA and PP5-shRNA, wounding of cells with PP5-shRNA caused a significant increase in p-Ser-FAK (Fig. 5B).

## Discussion

The early stages of wound repair in the lungs involves spreading and migration of epithelial cells to cover the denuded surface. This repair involves complex processes in which focal adhesion complexes alternately form and release attachments with the underlying substrate, and FAK has been shown to be a crucial component of protein complexes in focal adhesions^13, 14^. Using an approach in which we enriched the population of migrating cells in a multiple scratch wound model, we identified for the first time that CXCR4 and PP5 increased their interactions with FAK in migrating cells, while ASK1 dissociated from this complex. In addition, we showed that migrating cells exhibited decreased phosphorylation of ASK1 at Ser-966 and decreased serine phosphorylation of FAK.

FAK mediates cell migration through its involvement in membrane protrusion and turnover of focal adhesions, and the signaling pathways involved in these processes are complex and overlapping. Based upon our previous studies examining lung epithelial repair mechanisms^16–18, 23^ and other studies demonstrating interactions in progenitor B cells^24, 25^, we hypothesized that CXCR4 signaling interacted with FAK during cell migration, and our results demonstrated an increase in association between CXCR4 and FAK in a population of cells enriched in migrating cells (Fig. 1). CXCR4 is a ubiquitous chemokine receptor that is involved in the activation of actin polymerization and initiation of cell migration in different types of cells, including ATII cells^33–35^. We previously showed that both inhibition and decreased expression of CXCR4 led to significant inhibition of cell migration in scratch wound models^23^. The prior studies in B cells showed that CXCL12 promoted FAK translocation into lipid rafts^24, 25^, but these studies did not demonstrate the molecular interaction between FAK and CXCR4, either by co-localization or by immunoprecipitation. Because CXCR4 is a G-protein coupled receptor with seven transmembrane domain regions, the increased interactions between FAK and CXCR4 most likely occurs at the plasma membrane, but we did not determine whether this occurs in lipid rafts or other regions.

In addition to the interaction between FAK and CXCR4, our immunoprecipitation studies showed basal interactions between FAK, CXCR4, PP5, and ASK1 in quiescent (unwounded) cells, but increased interactions among FAK, CXCR4, and PP5, and decreased interactions with ASK1, in migrating cells following wounding (Fig. 1). These results suggest increased assembly of a complex containing FAK, CXCR4, and PP5 that promotes cell migration and repair, with a corresponding loss of ASK1 from the complex. In support of these findings, and to demonstrate functional relevance, knockdown of PP5 using shRNA significantly inhibited cell migration, while ATII cells from ASK1−/− mice exhibited accelerated wound closure (Fig. 2). Furthermore, knockdown of ASK1 by shRNA or deficiency in ASK1−/− cells significantly increased the association between FAK and CXCR4 in unwounded cells (Fig. 3). In contrast, knockdown of PP5 had no effect on FAK-CXCR4 interactions (Fig. 3A). While the formation of complexes with FAK, integrins, and other signaling molecules in migrating cells has been extensively studied, there are no previous reports of interactions between FAK, CXCR4, PP5, and ASK1 regulating cell migration.

ASK1 is ubiquitously expressed and is known to regulate p38- and JNK-mediated inflammation and apoptosis^36, 37^. Because our previous studies demonstrated FAK-dependent activation of JNK in migrating epithelial cells^18^, we hypothesized that ASK1 was involved in the regulation of JNK-mediated motility. In our studies with lung epithelial cells, deficiency of ASK1 stimulated cell migration, with a corresponding increase in interactions between FAK and CXCR4 (Figs 2 and 3). Inhibition of ASK1 was previously shown to inhibit cell motility in both oral squamous carcinoma cells^27^ and chondrosarcoma cells^28^, but knockdown of ASK1 in breast cancer cells stimulated cell migration^26^. Thus, the regulatory role of ASK1 in cell migration appears to vary among cells from different tissues, similar to its tissue-specific regulation of inflammation and apoptosis. A limitation of our approach is that we have interpreted wound closure after 24 hr as an index of primarily cell spreading and migration, but cell proliferation can also contribute to wound closure over longer periods of time. Our previous measurements with primary rat ATII cells showed that only ~13% of cells near the wound edge had incorporated ethidium deoxyuridine (as an index of proliferation) by 24 hr^38^, suggesting a relatively small contribution of proliferation to wound closure at that time.

ASK1 interacts with a significant number of intracellular proteins with an extensive range of functions, and the term ASK1 signalosome has been used to describe a high molecular mass complex (>1500 kDa) that forms in cells^39, 40^. ASK1 also contains multiple phosphorylation sites that regulate function, activity, and association with other proteins^39, 41^. Activation of ASK1 is reported to be regulated by phosphorylation of a threonine residue (Thr^838^ for human, Thr^845^ for mice) within the kinase domain. In addition phosphorylation of serine residues Ser^83^, Ser^966^, and Ser^1033^ cause association of regulatory molecules that result in inactivation of ASK1. We found that wounding caused a significant decrease in the content of p-Ser^966^-ASK1 in cells (Fig. 4B), and this corresponded with a significant decrease in p-Ser^966^-ASK1 associated with FAK (Fig. 4C). In contrast the content of p-Ser^83^-ASK1 was unaffected by wounding (data not shown). Due to the limitations of available antibodies, we did not determine whether p-Ser^1033^-ASK1 was affected by wounding. These results suggest that dephosphorylation of ASK1 at Ser^966^ in wounded cells promotes dissociation of ASK1 from the FAK/CXCR4/PP5 complex. The change in phosphorylation may lead to changes in conformation and activation of ASK1. For example, phosphorylation of Ser^966^ has been shown to facilitate the association of ASK1 with the 14-3-3 family of proteins and subsequent decreased ASK1 activity^42–44^. We did not determine whether 14-3-3 proteins were part of the FAK/CXCR4/PP5/ASK1 complex. The purpose of ASK1 dissociation and the mechanisms by which this promotes cell migration remain to be determined.

In addition to the changes in ASK1 phosphorylation following wounding, we also detected a decrease in p-Ser on FAK using the 1C8 antibody. FAK contains 25 sites for phosphorylation, including 15 serine, 5 threonine, and 5 tyrosine residues^32^. Conformation changes in FAK following tyrosine phosphorylation at different sites has been shown to regulate integrin engagement and interactions with other signaling molecules, notably the creation of high affinity binding sites following phosphorylation at Tyr^397^ (reviewed in refs 15, 45–47). As stated above there are few studies on the role of serine phosphorylation of FAK^31, 32^. We used a phosphoserine detection kit to investigate changes in serine phosphorylation on FAK following wounding. Of the panel of antibodies in the kit, only the 1C8 monoclonal antibody indicated a difference in p-Ser-FAK following wounding (Fig. 4A). However, we do not know at this time which of the 15 serine sites on FAK exhibited this change in phosphorylation.

Because we observed loss of serine phosphorylation on both FAK and ASK1 we investigated whether PP5 regulated these events. PP5 is a member of the serine/threonine phosphatase family of proteins, and has been shown to be involved in numerous cellular processes, including MAPK-mediated signaling^48, 49^. PP5 has been reported to interact with ASK1 and cause dephosphorylation at Thr-845^29, 50^. We found that shRNA-induced knockdown of PP5 caused decreased p-Ser^966^-ASK1 in basal cells (Fig. 5A). In contrast with control cells, wounding did not cause a decrease in p-Ser^966^-ASK1 in cells deficient in PP5. Thus, PP5 may regulate the baseline association of ASK1 within the complex, but it does not appear to regulate its dissociation following wounding of cells. There is some evidence that PP2B may also regulate phosphorylation of ASK1^51^, but we did not investigate PP2B in this study. Wounding also caused a decrease in p-Ser-FAK phosphorylation in rat ATII cells (Fig. 4A). While we did not observe a significant decrease in p-Ser-FAK in control MLE-12 cells following wounding (Fig. 5B), we did see a significant increase in p-Ser-FAK following wounding of MLE-12 cells with PP5-shRNA knockdown. These results suggest that PP5 regulates the phosphorylation of FAK at the serine site recognized by the 1C8 antibody. Further analysis using antibodies with greater specificity and mass spectrometry will be necessary to specifically identify which site on FAK is regulated and whether there are other phosphatases involved.

The changes in serine phosphorylation on ASK1 and FAK, and the regulation by PP5, point toward an important role for serine kinases in the regulation of cell migration. It is well-established that phosphoinositide-3 kinase (PI-3 kinase) and Akt promote cell migration through serine phosphorylation, including in bronchial epithelial cells^52, 53^. Earlier studies also demonstrated that activation of protein kinase A accelerates bronchial epithelial cell wound closure^54^, while activation of protein kinase C inhibits wound closure^55, 56^. While these two serine/threonine kinases may undergo bi-directional regulation, these processes may also be regulated in part by protein phosphatases. We did not investigate the regulation of these kinases in the context of the molecular complex described above.

In summary, our results reveal novel interactions among FAK, CXCR4, ASK1, and PP5 that regulate cell migration following wounding. Under quiescent conditions, these molecules are associated with one another, as well as other proteins that may be part of the complex. Following injury to the epithelial cells, there is greater association between FAK, CXCR4, and PP5, but ASK1 dissociates from the complex in migrating cells. The dissociation of ASK1 corresponds with a decrease in phosphorylation of ASK1 at Ser^83^ and a decrease in serine phosphorylation of FAK at a site recognized by the 1C8 antibody. These molecular interactions may be important in the repair of the epithelium following injury to the lungs due to their role in efficient cell migration. In this context, acute or repetitive injuries that disrupt these signaling pathways and molecular interactions could result in dysfunctional repair, and conversely, promotion of these pathways and interactions could promote repair.

## Methods

### Reagents

Dulbecco’s Modified Eagle’s Medium (DMEM), penicillin-streptomycin, trypsin-ethylenediaminetetraacetic acid (EDTA) solution, and phosphate buffer saline were purchased from GIBCO Life Technologies (Grand Island, NY). Fetal bovine serum (FBS) was obtained from Hyclone (Logan, UT). *4-*(*2-hydroxyethyl*)*-1-piperazineethanesulfonic acid* (*HEPES*) was purchased from Gibco (Grand Island, NY). Tween-20 was purchased from Bio-Rad (Hercules, CA). Gradient gels (4–12%) for Western blot were purchased from Invitrogen Inc. (Carlsbad, CA). Antibodies against CXCR4 were purchased from abCAM (Cambridge, MA, cat. #ab13854). Antibodies against focal adhesion kinase-1 (FAK) were purchased from BD-Bioscience (San Jose, CA). Antibodies against protein phosphatase-5 (PP5) were purchased from Santa Cruz Biotech (Santa Cruz, CA). Antibodies against apoptosis signal regulating kinase-1 (ASK1) were purchased from Genetex Inc. (Irvine, CA). A monoclonal antibody against phophoserine (clone 1C8) was purchased as part of a phosphoserine detection kit from EMD Millipore (San Diego, CA, cat. #525282).

### Cell culture

Primary rat ATII cells were isolated according to the methods described previously^57, 58^. The animal use protocol was approved by the Institutional Animal Care and Use Committee at the University of Tennessee Health Science Center. Briefly, ATII cells were isolated from male Sprague-Dawley rats by elastase digestion and differential adherence on IgG-coated dishes. ATII cells were identified using Nile Red (Sigma) staining of lamellar bodies, and >95% of the cells were Nile Red-positive on *day 2*. We used plastic 6-well plates or chamber slides that were coated with rat lung fibroblast (RFL) matrix deposited by RLF-6 cells (American Type Culture Collection) for wound healing assays with ATII cells^57^. Freshly isolated cells were seeded to confluence at 3.5 × 10^6^ cells/well in ATII culture medium (DMEM with 10% FBS, 4 mM glutamine, 1% penicillin/streptomycin, and 0.25 µM amphotericin B), and experiments were performed on *day 2* after isolation. MLE-12 cells, a transformed cell line of mouse alveolar epithelial cells^59^, were cultured using media containing DMEM with 10% FBS, 4 mM glutamine, 1% penicillin/streptomycin.

### Isolation of AT2 cell from wild type and ASK1-Knockout mice

ATII cells from wild type and ASK1 knockout mice (ASK1−/−) were isolated according to previous methods^60^. The yield per mouse lung was 3 to 5 million with >90% purity assessed by immuno-staining for surfactant protein-C. The animal use protocol for these studies was approved by the Institutional Animal Care and Use Committee at the University of Tennessee Health Science Center (UTHSC). All experiments were performed in accordance with UTHSC guidelines and regulations.

### Development of ASK1 and PP5-shRNA cell lines

*S*table cell lines with decreased expression of ASK1 and PP5 were developed in A549 (ASK1) and MLE-12 (PP5) cells using shRNA (SantaCruz Biotech, ASK1-Cat# sc-29749-V and PP5-Cat# sc-44603-V) according to the manufacturer’s protocol. A cell line with control shRNA was developed using a scrambled vector (SantaCruz Biotech, Cat# SC-108080). The antibiotic puromycin was used in the media to select the positive clone. The knockdown status was assessed by western blotting for protein expression.

### Wound healing assay

Cell migration was measured according to our previous methods^61^. Confluent monolayers of ATII, A549, MLE12, or shRNA knockdown cells were wounded by scraping multiple pipette tips across the monolayer to produce initial wounds of 1000–1,200 µm. Images were collected with a Cool Snap charge-coupled device camera (Roper Scientific, Trenton, NJ) mounted on an Eclipse TE300 inverted microscope with a 4X objective (Nikon, Melville, NY). Images were obtained at the initial time of wounding and then 24 hrs post-wounding. Metamorph software (Universal Imaging, Westchester, PA) was used to record the coordinates for each wound location using a computer-controlled stage so that the same location was used for post-wounding measurement, and data were analyzed by Metamorph imaging software. The mean wound width at 24 hrs was calculated and normalized to the original wound width as a percentage of the original width. In some studies we used the IncuCyte ZOOM imaging system (Essen Bioscience, Ann Arbor, MI) to measure wound closure. Scratch wounds were created in all 96 wells of a plate using the Essen Bioscience Woundmaker, and images were captured every 120 minutes and analyzed using Metamorph as described above. All scratch wound results reported are from at least three independent wells from at least two separate experiments (*n* = 6).

### Immunoprecipitation and immunoblotting

Confluent monolayers of cells were wounded using a multi-pronged comb so as to generate a large number of wound edges for each well, and after 24 hr of cell migration the cells were lysed using RIPA buffer. Unwounded monolayers were used as controls. The cell lysates were then subjected to immunoprecipitation using antibodies against FAK, CXCR4, PP5, and ASK1. Protein concentration was determined by the Bradford method^62^. Equal amounts of protein were resolved by 4–12% SDS-PAGE, and electrophoretically transferred onto PVDF membranes. Membranes were blocked for 1 h in 5% nonfat milk (Bio-Rad, Hercules, CA) in Tris buffered saline (TBS) containing 0.01% Tween 20 (TBST) and incubated overnight at 4 °C with the appropriate primary antibody. Membranes were washed with TBST and then incubated with the secondary antibody for 1 h at room temperature. Blots were developed on X-ray film using the enhanced chemiluminescence method (ECL) (Amarsham Biosciences, Piscataway, NJ). Reciprocal IP and immunoblot experiments were performed 2 to 4 times for each result shown.

### Densitometry of immunoblot

Densitometry of bands obtained by immunoblot was performed using the software Image-J developed by the National Institutes of Health (NIH). Each band was normalized against the corresponding beta-actin, GAPDH, or total expression. These values were then normalized against the control condition.

### Statistical analysis

All values are presented as means ± standard error (SE). A *t*-test was used when only two groups were compared, and one-way ANOVA with the Holm-Sidak method was performed for comparisons of multiple treatments to determine significant differences between individual conditions. Significant differences were determined based on a threshold of p < 0.05. Statistical comparisons were made using Sigmastat 3.5 (Jandel Scientific, San Rafael, CA).

## Electronic supplementary material

Supplemental figures R1
