# Supplementary material for: A heteromeric molecular complex regulates the migration of lung alveolar epithelial cells during wound healing
Source: Sci Rep. 2017 May 19;7:2155. doi: 10.1038/s41598-017-02204-2 (PMC5438388; doi:10.1038/s41598-017-02204-2)

Supplemental Data

A heteromeric molecular complex regulates the migration of lung alveolar epithelial cells during wound healing

Manik C. Ghosh[1], Patrudu S. Makena[2], Joseph Kennedy[1], Bin Teng[1], Charlean Luellen[1], Scott E. Sinclair[1,2], and *Christopher M. Waters[1,2]

Departments of [1]Physiology and [2]Medicine, University of Tennessee Health Science Center, Memphis, TN, 38163

Fig. 1

<u>IP: FAK1</u> WB: Ask1

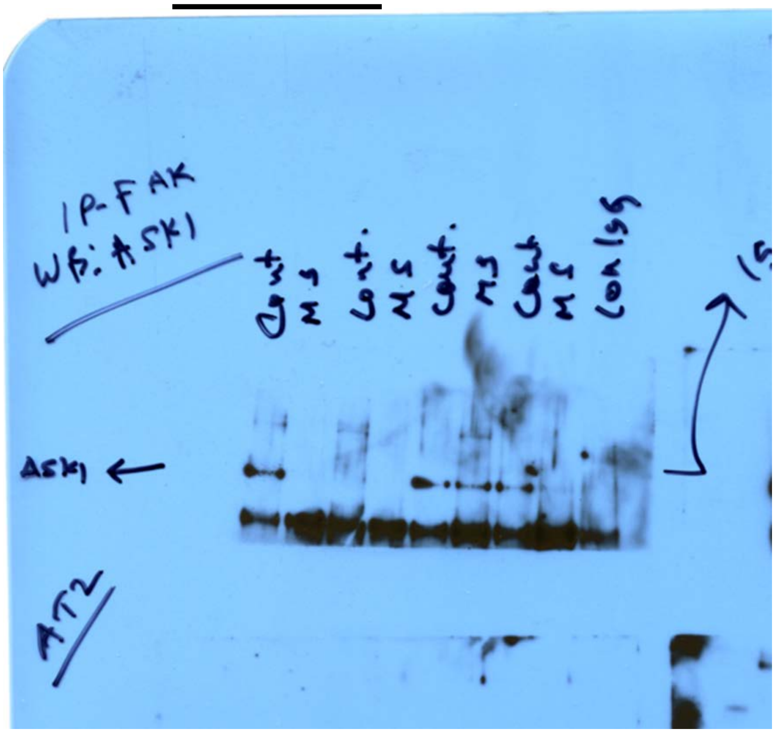

<u>IP: FAK1</u> WB: CXCR4

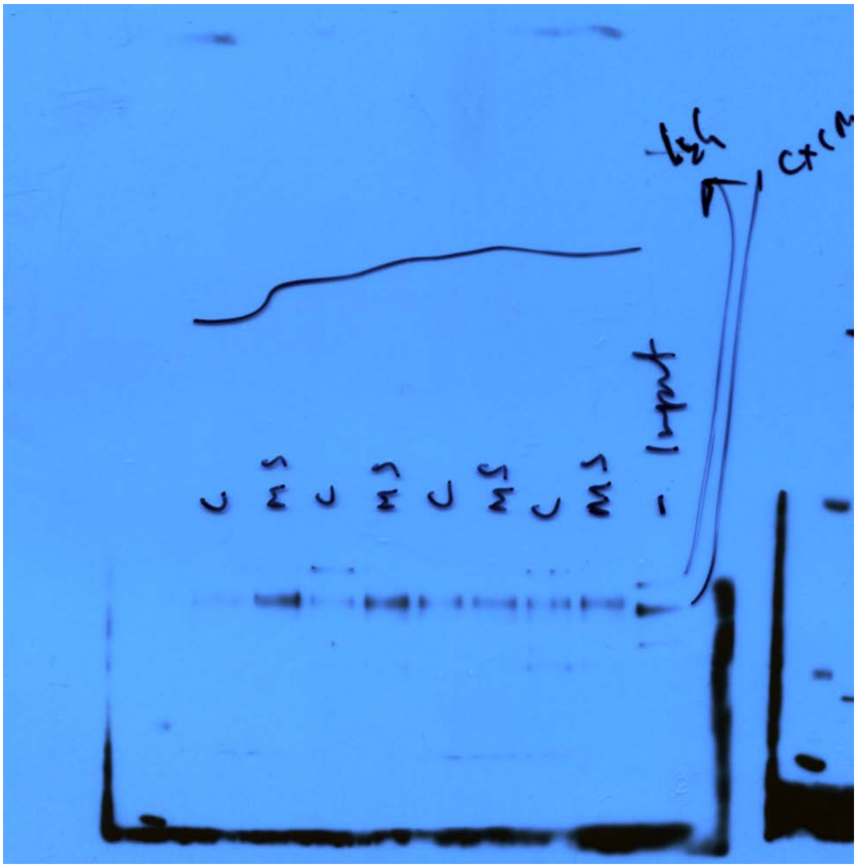

Fig. 1

IP: FAK1 WB: PP5

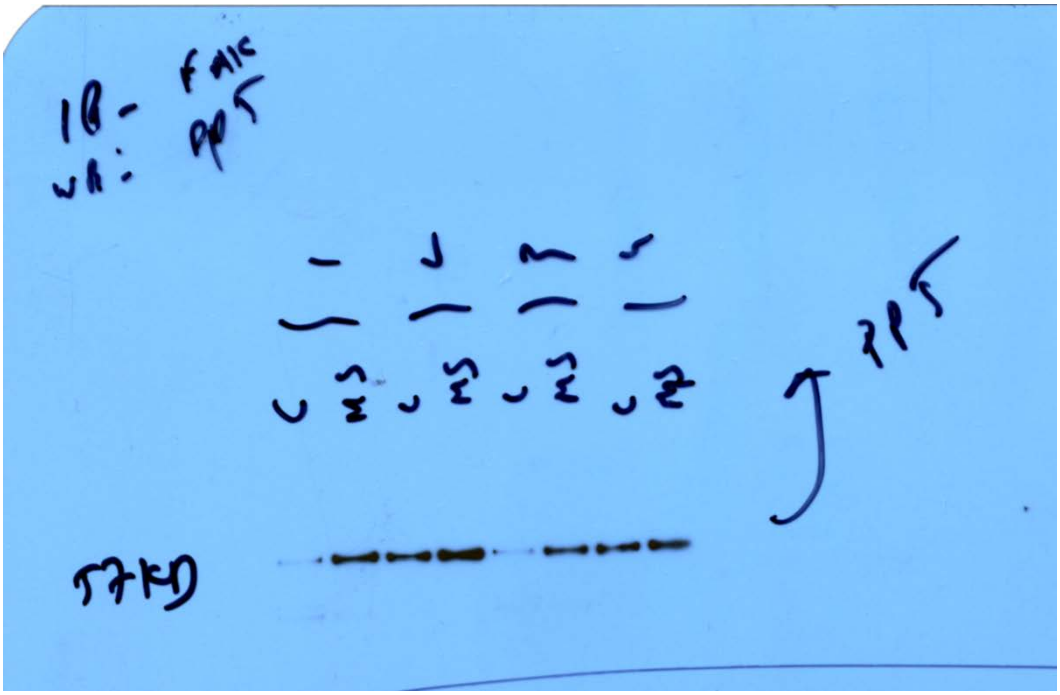

IP: FAK1 WB: FAK1

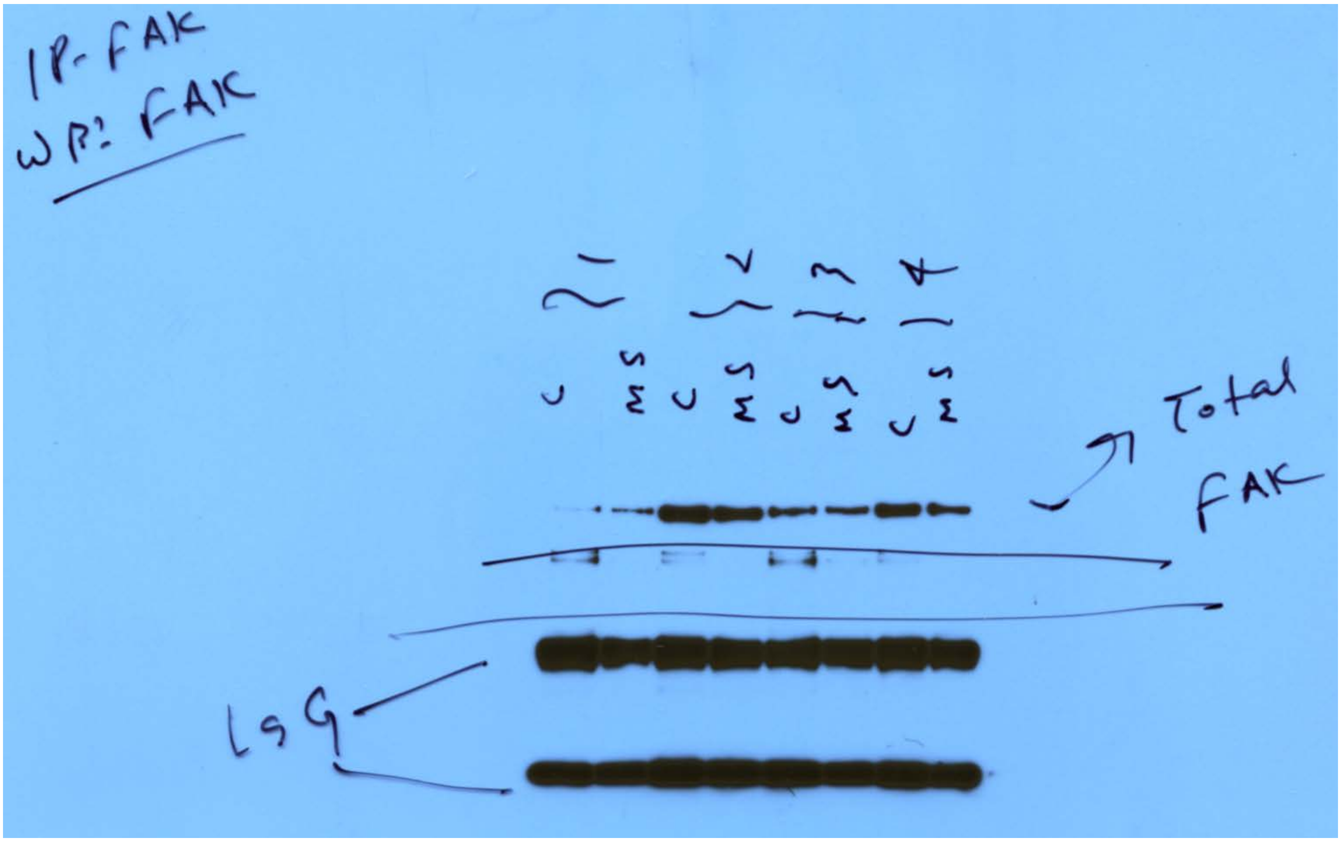

Fig. 1

<u>IP: PP5</u> WB: Ask1

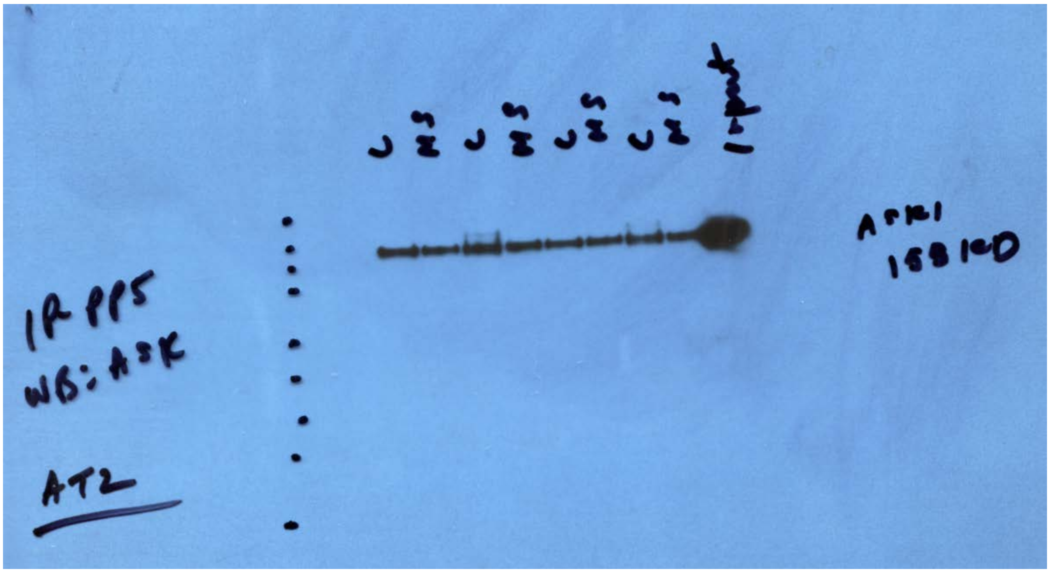

<u>IP: PP5</u> WB: CXCR4

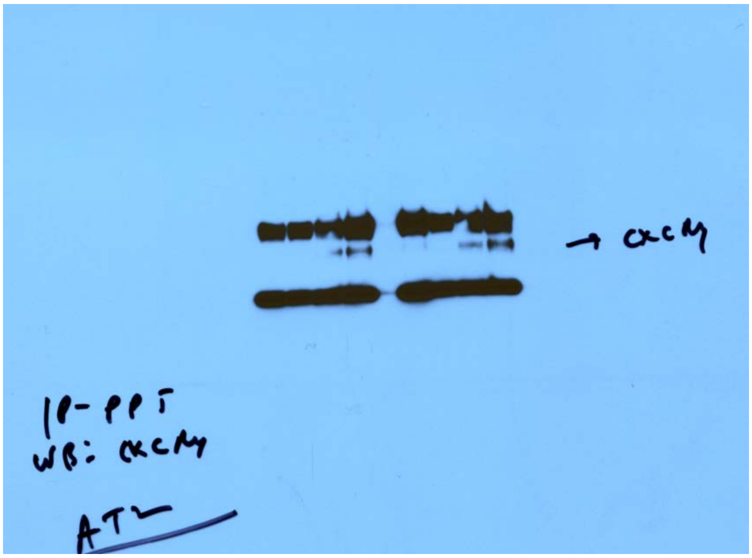

Fig. 1 **<u>IP: PP5</u> WB: FAK1**

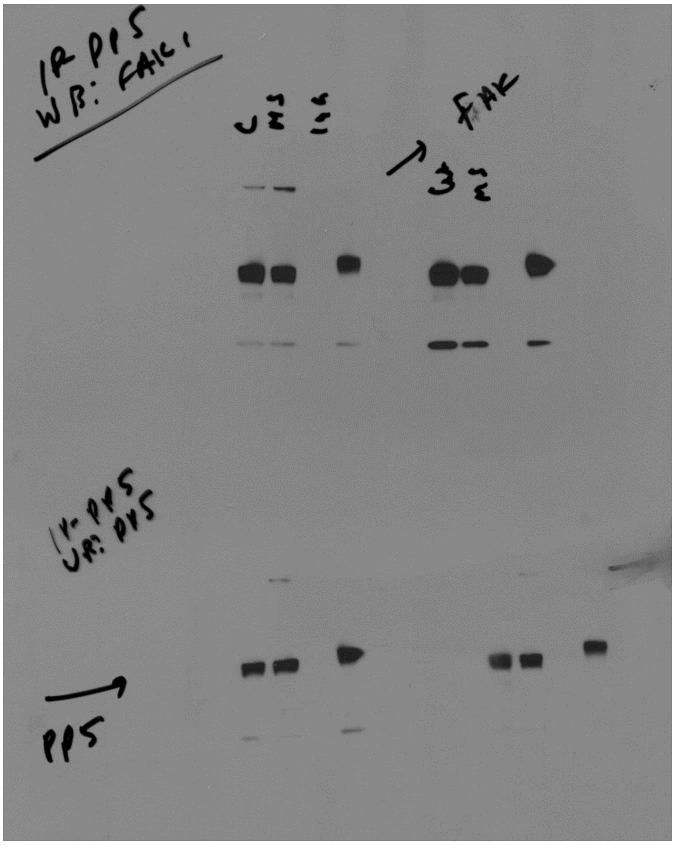

<u>IP: PP5</u> WB: PP5 (same film)

Fig. 1

IP: CXCR4 WB: ASK1

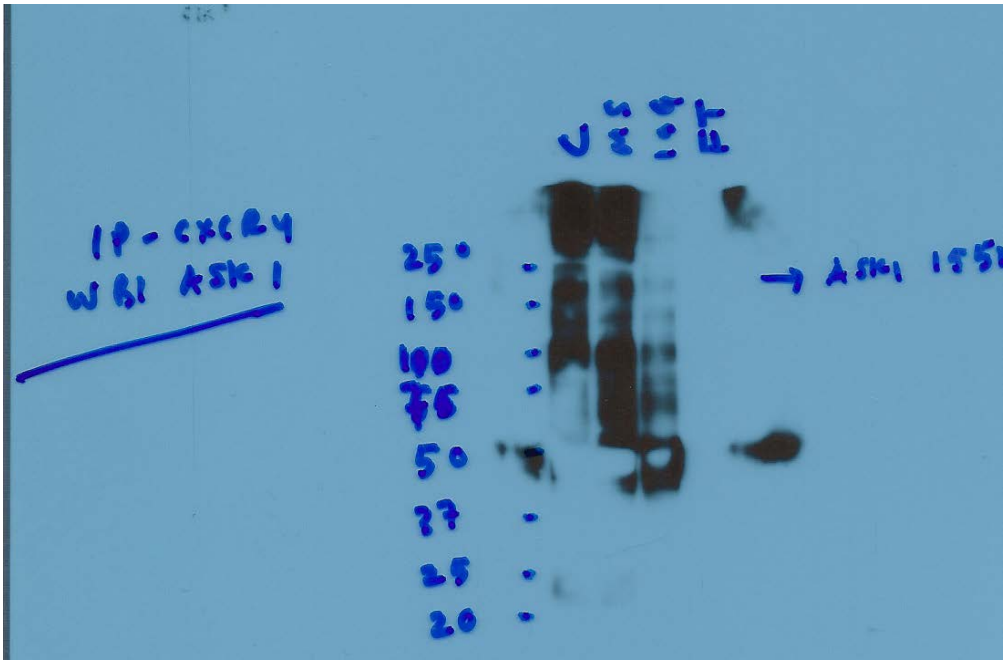

IP: CXCR4 WB: CXCR4 **IP: CXCR4 WB: FAK1**

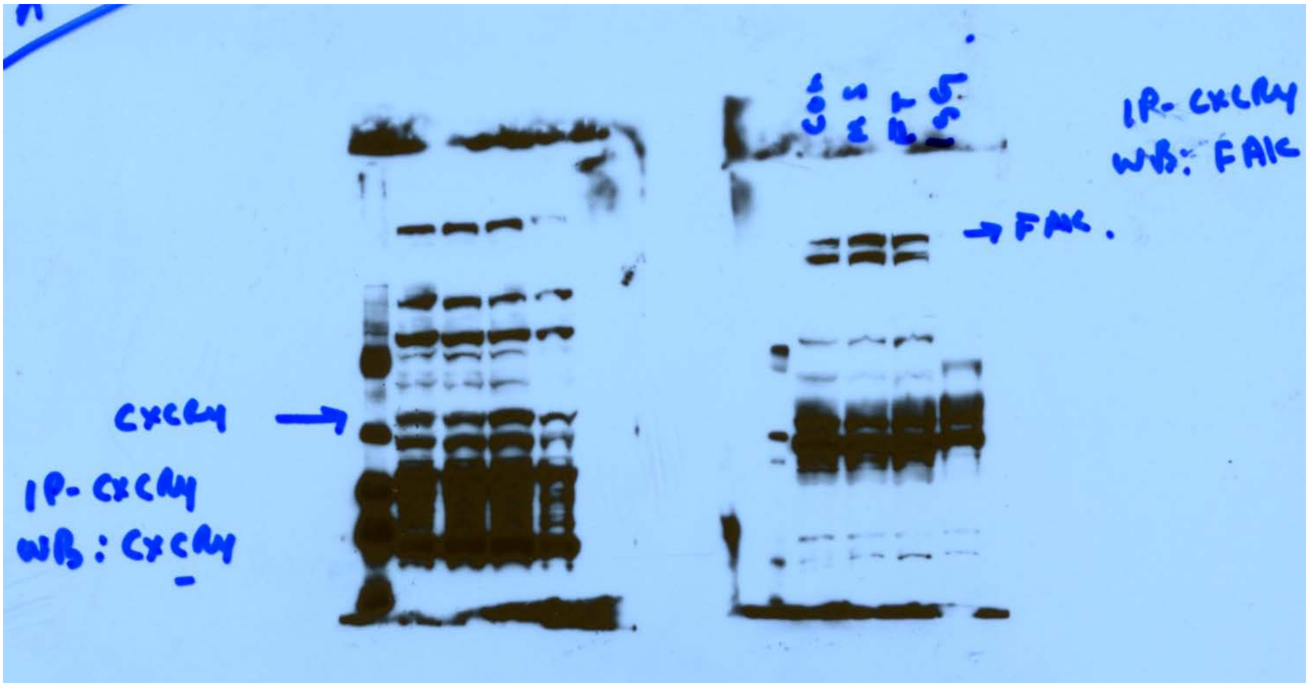

Fig. 1 **IP: ASK1 WB: CXCR4**

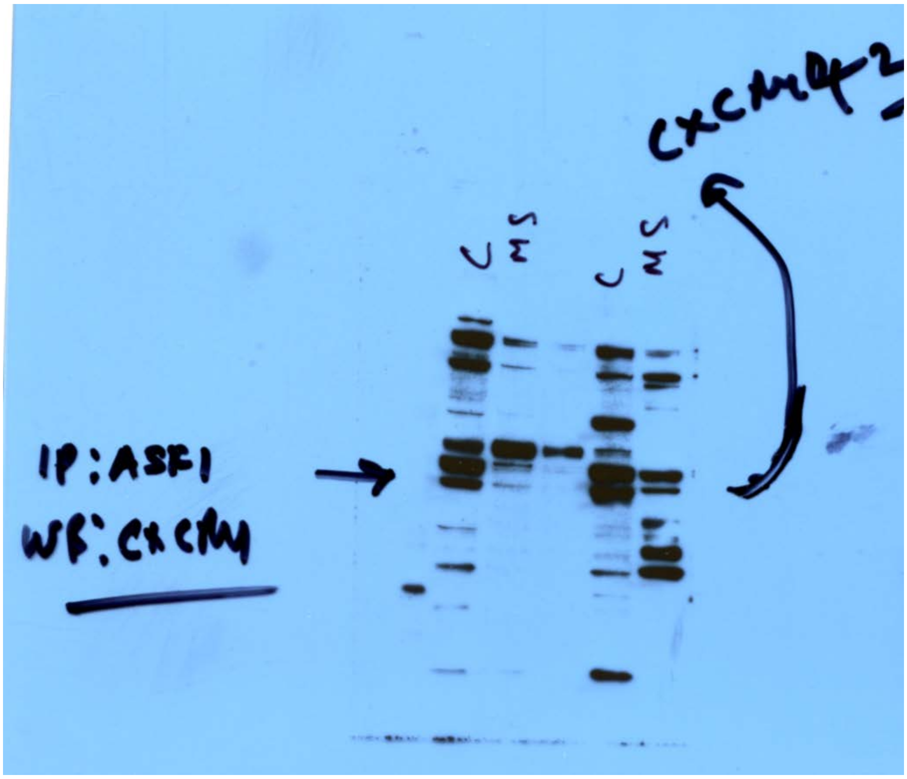

IP: ASK1 WB: FAK1

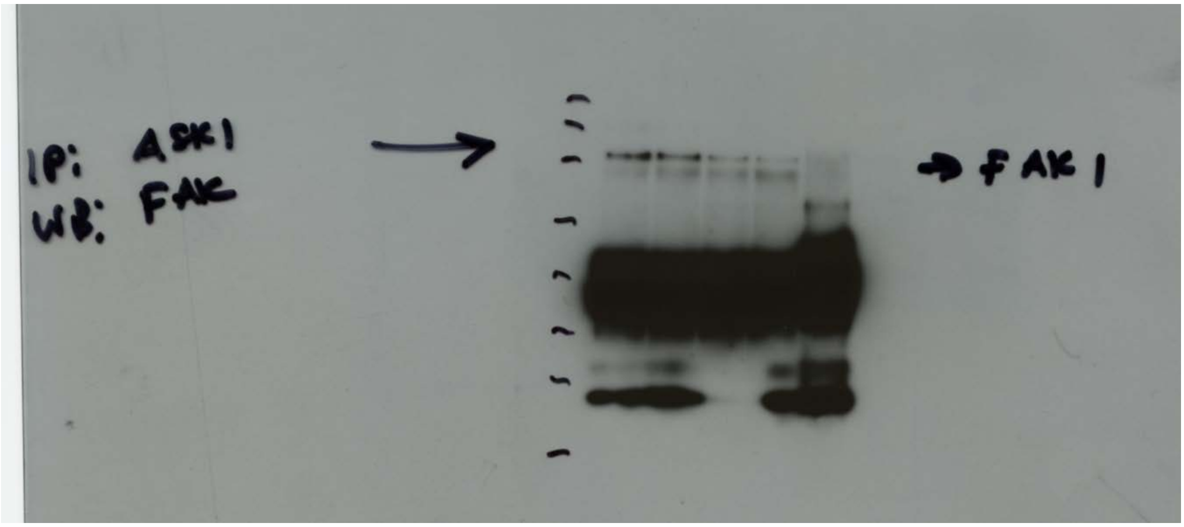

Fig. 1

<u>IP: ASK1</u> WB: ASK1

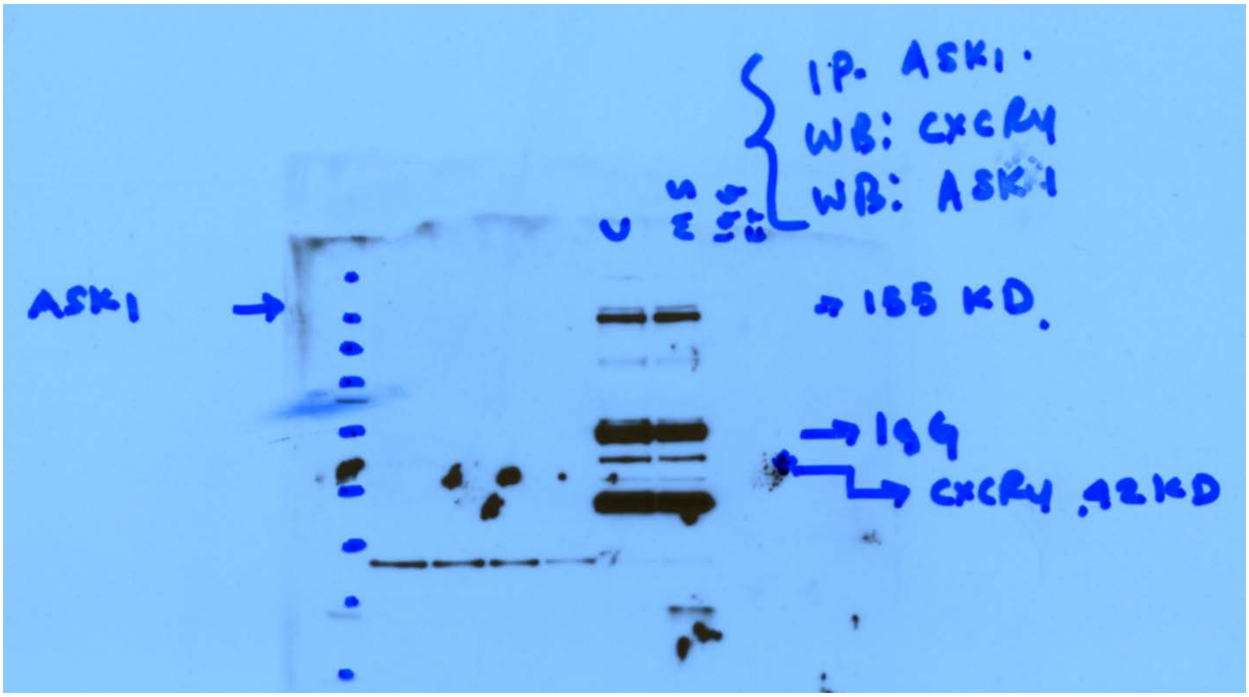

Fig. 2A

WB: ASK1 and b-actin

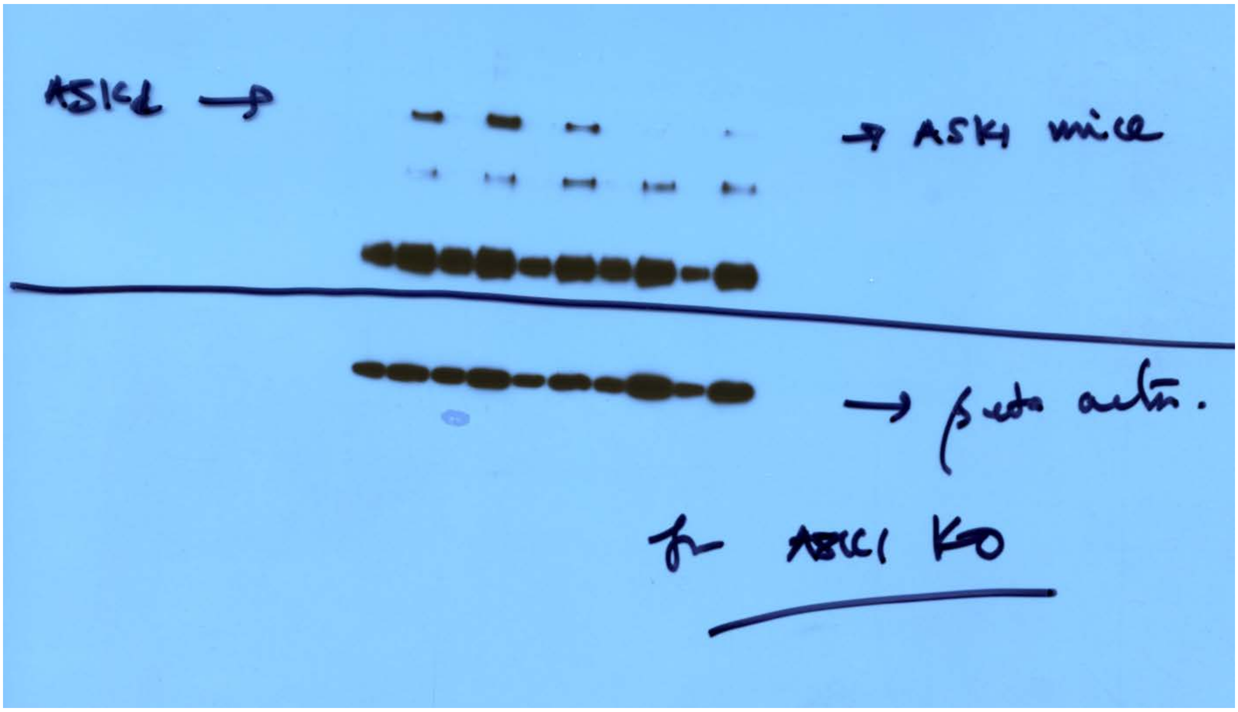

Fig. 2B

WB: PP5 and b-actin

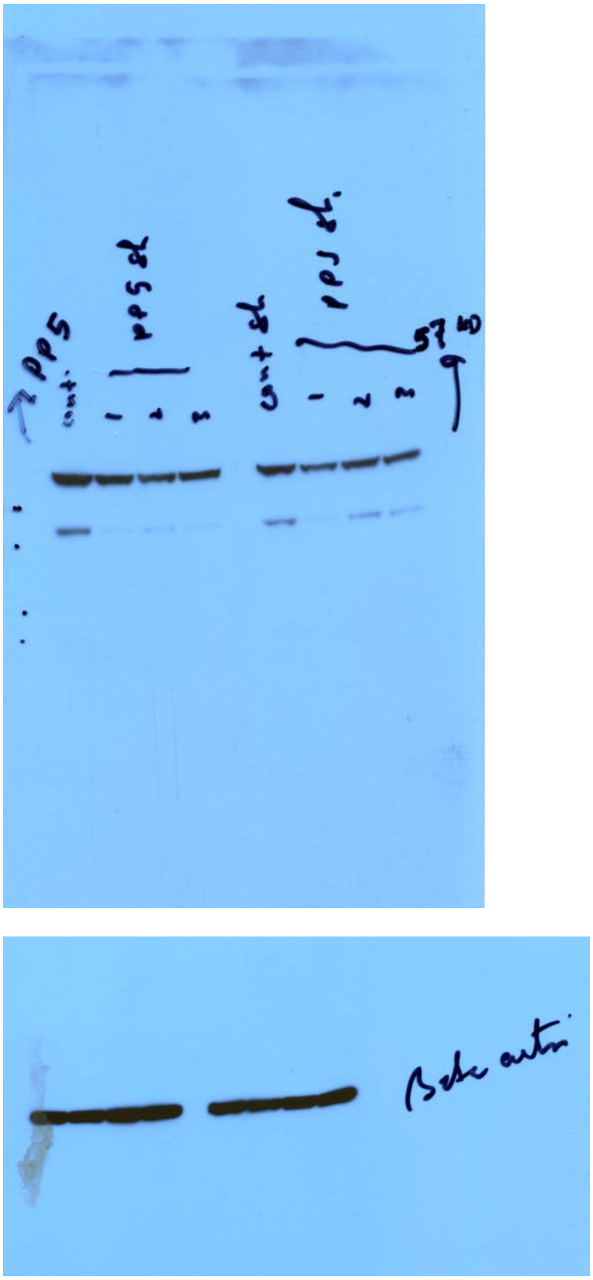

Fig. 3A

IP: FAK WB: CXCR4

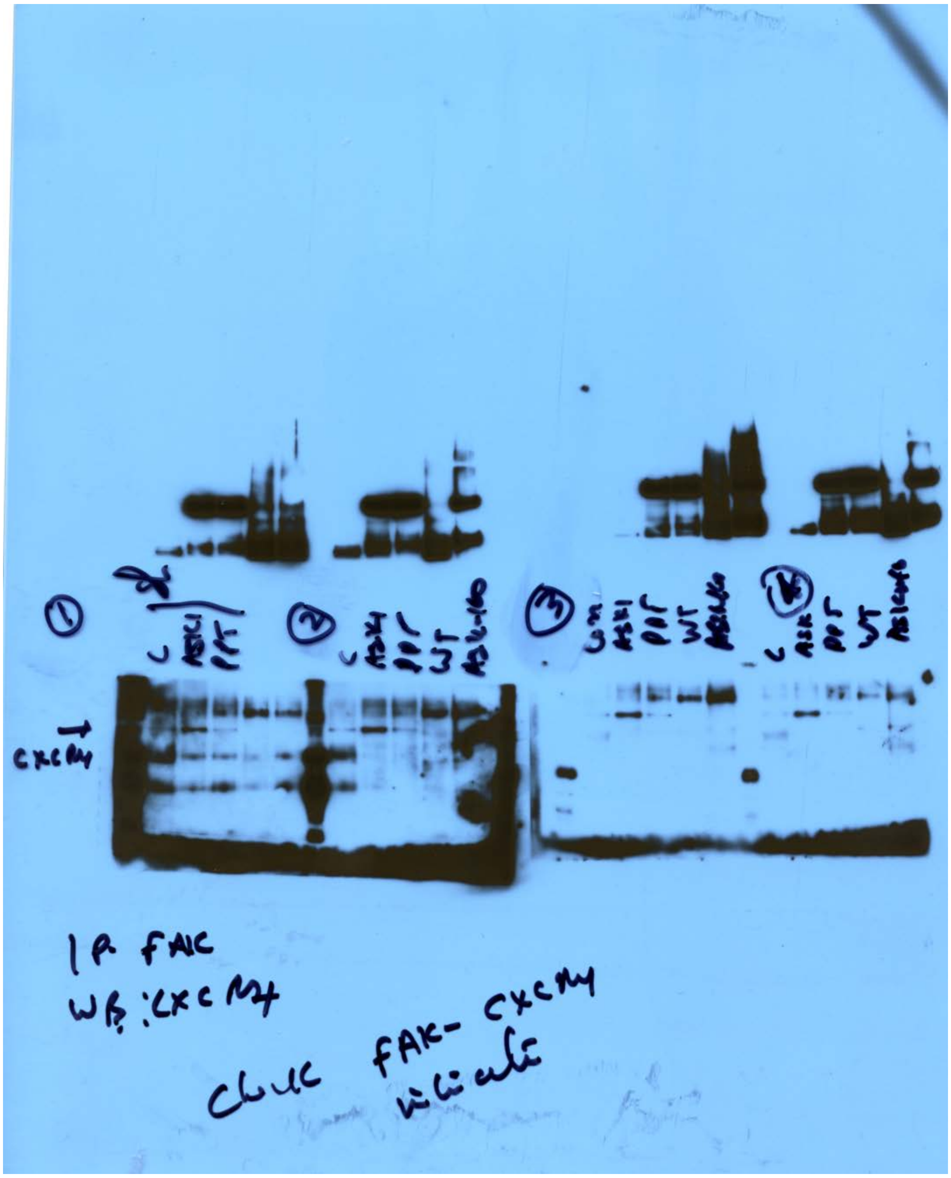

Fig. 3A

<u>IP: FAK</u> WB: FAK

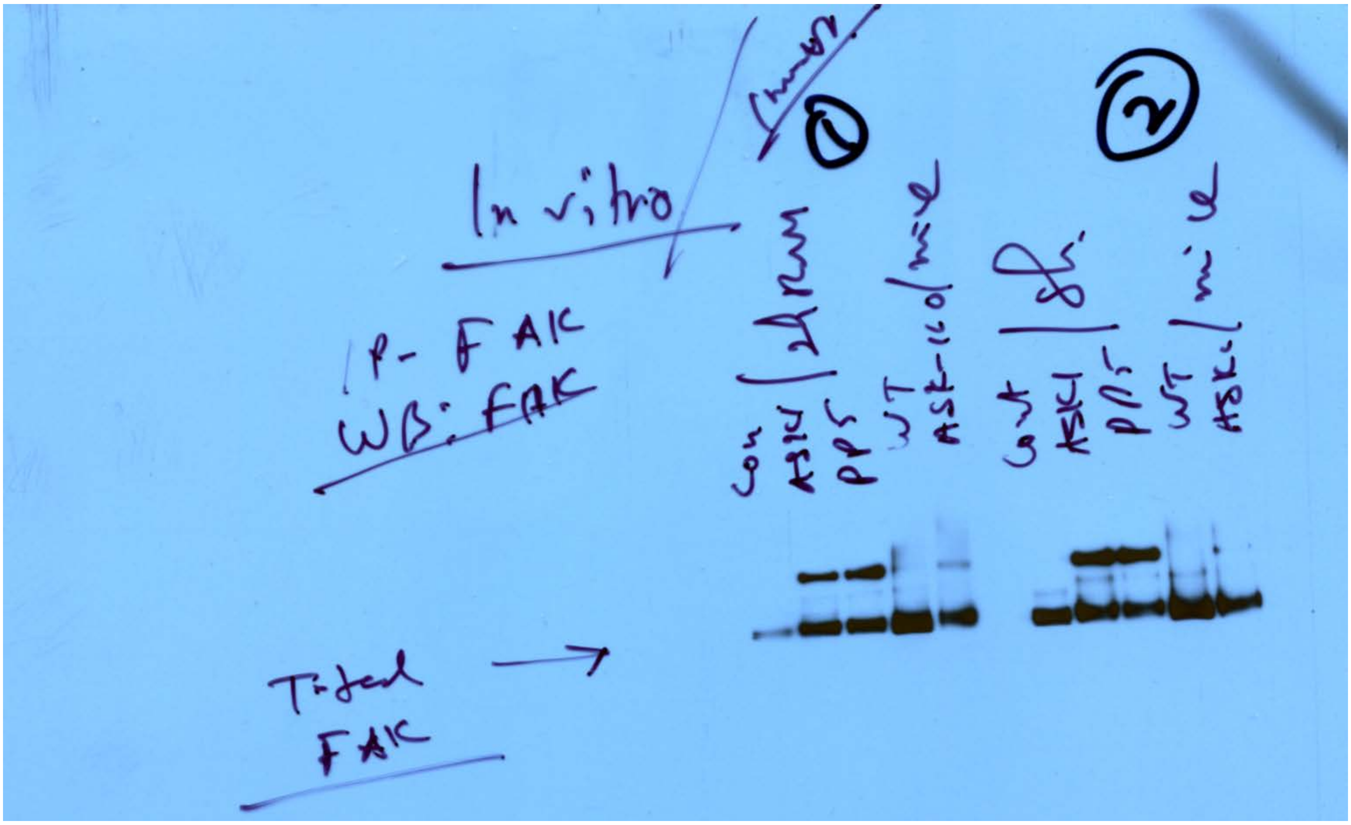

Fig. 3B

<u>IP: FAK</u> WB: CXCR4

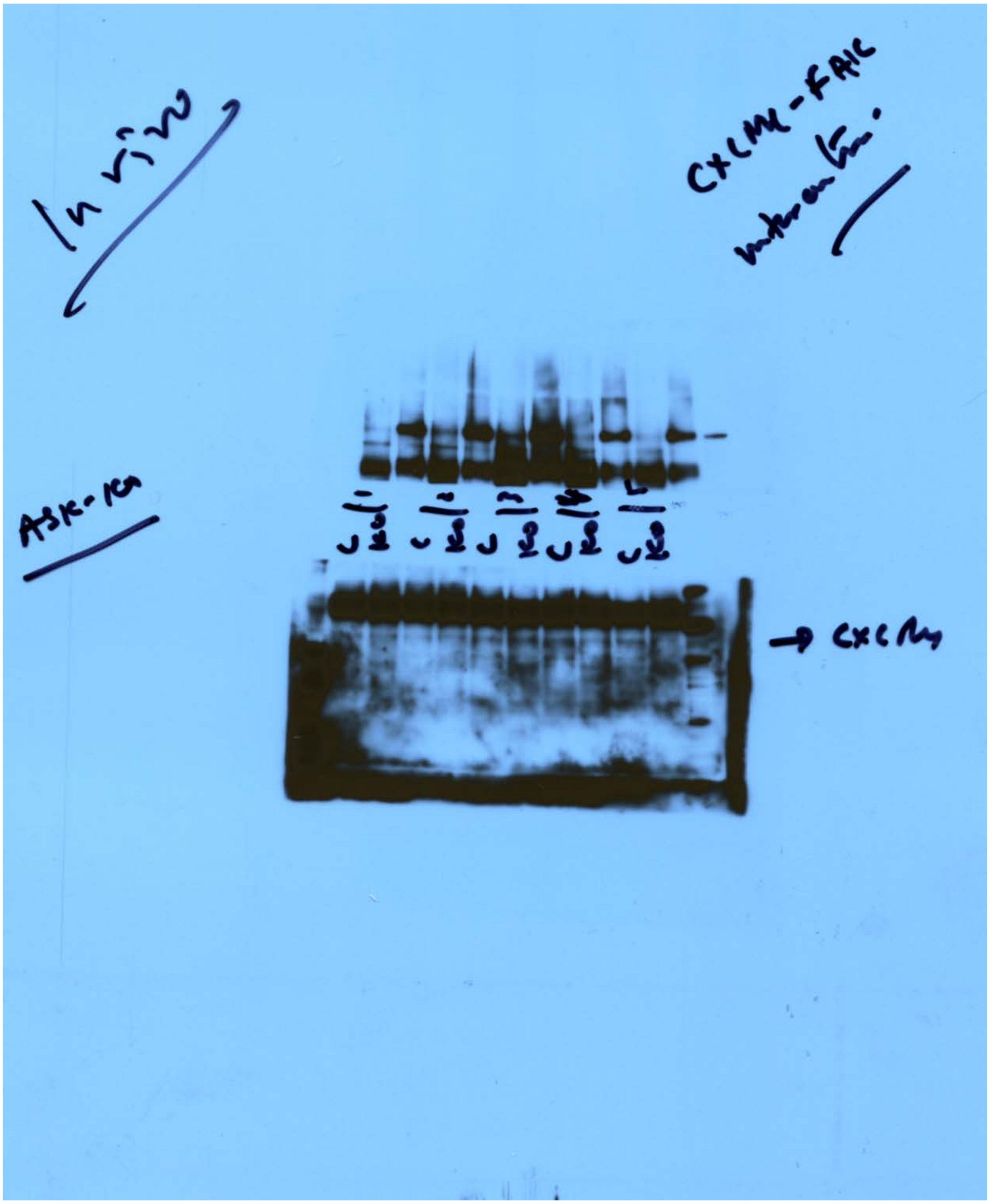

Fig. 3B

<u>IP: FAK</u> WB: FAK

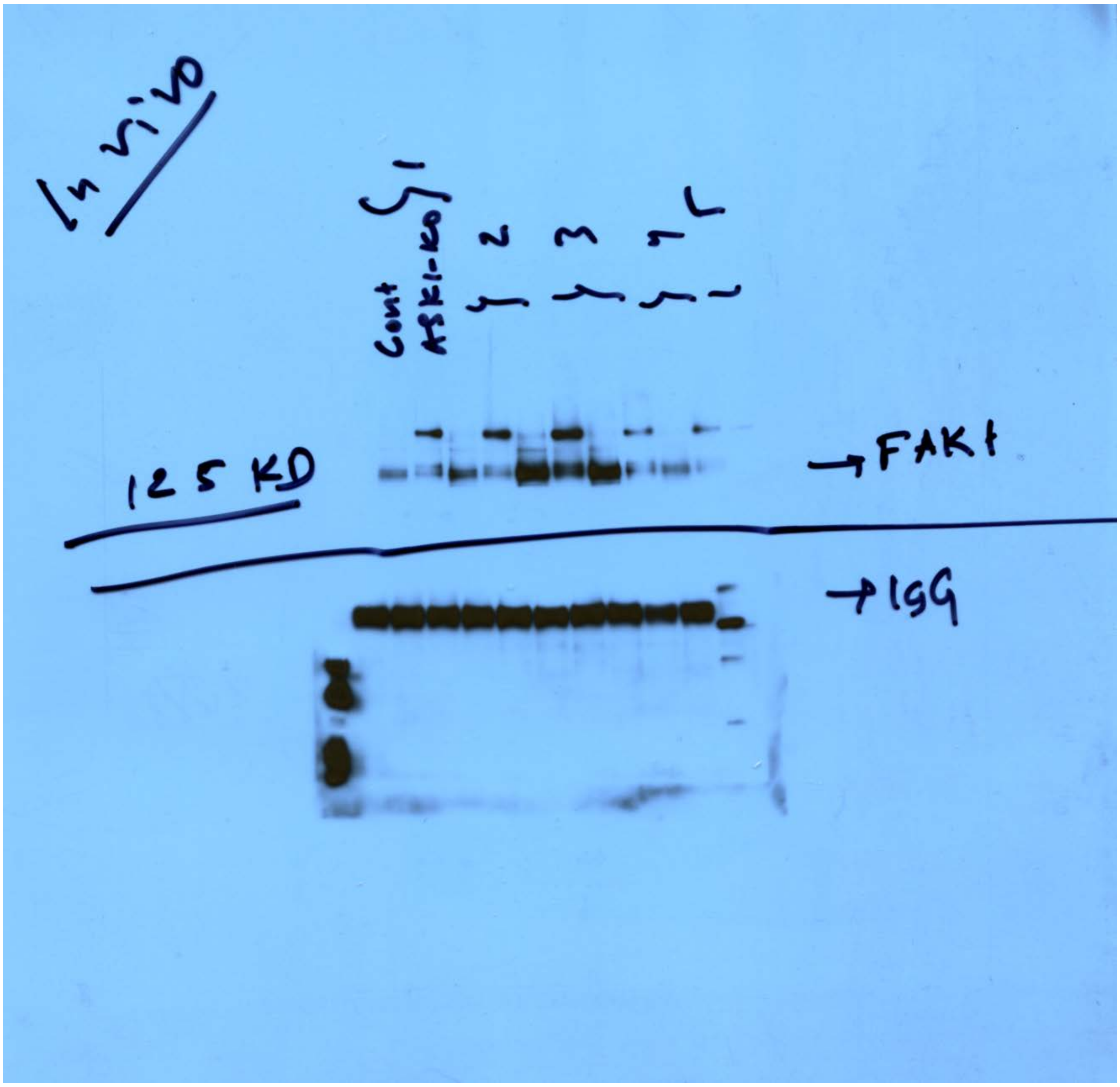

Fig. 4A

IP: FAK WB: p-Ser (1C-8)

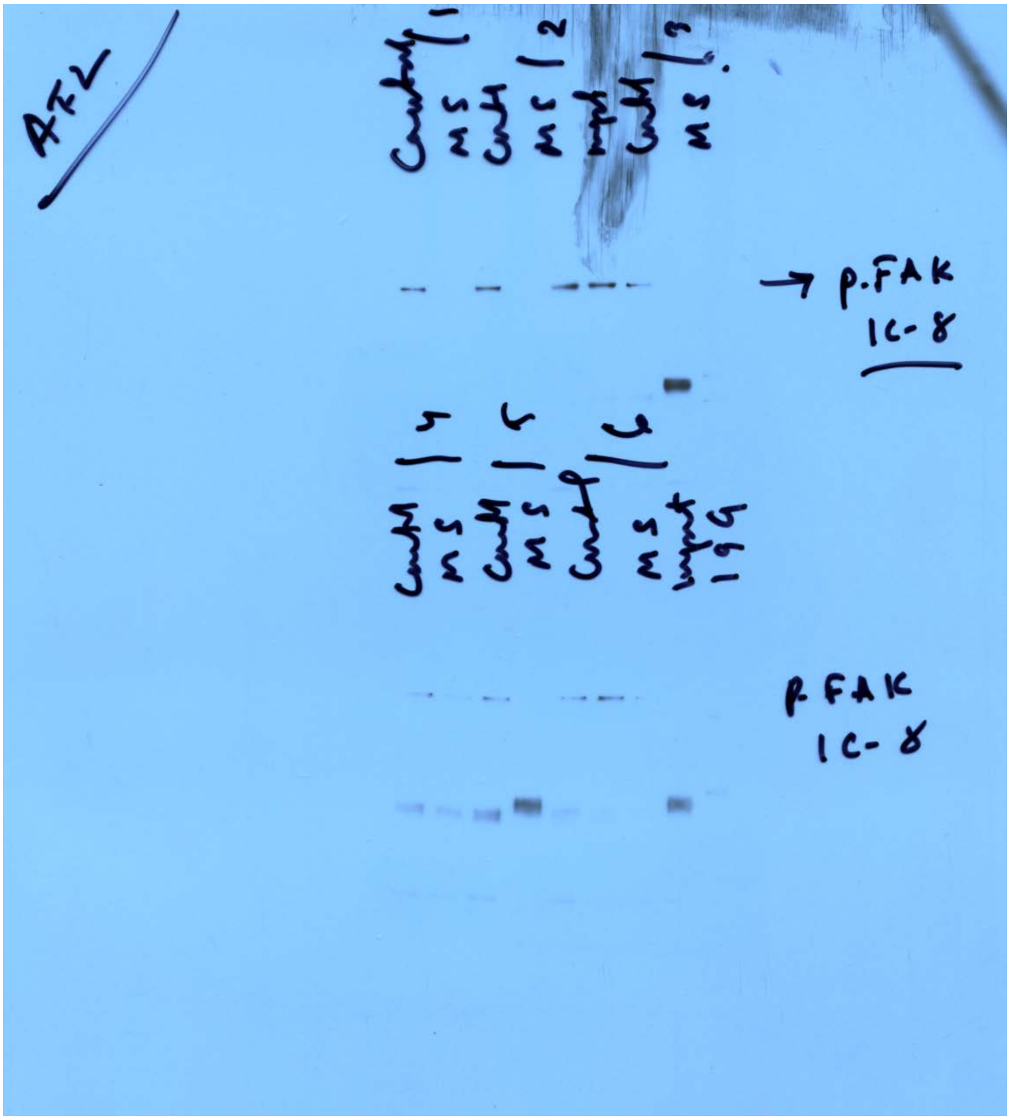

Fig. 4A **<u>IP: FAK</u> WB: FAK**

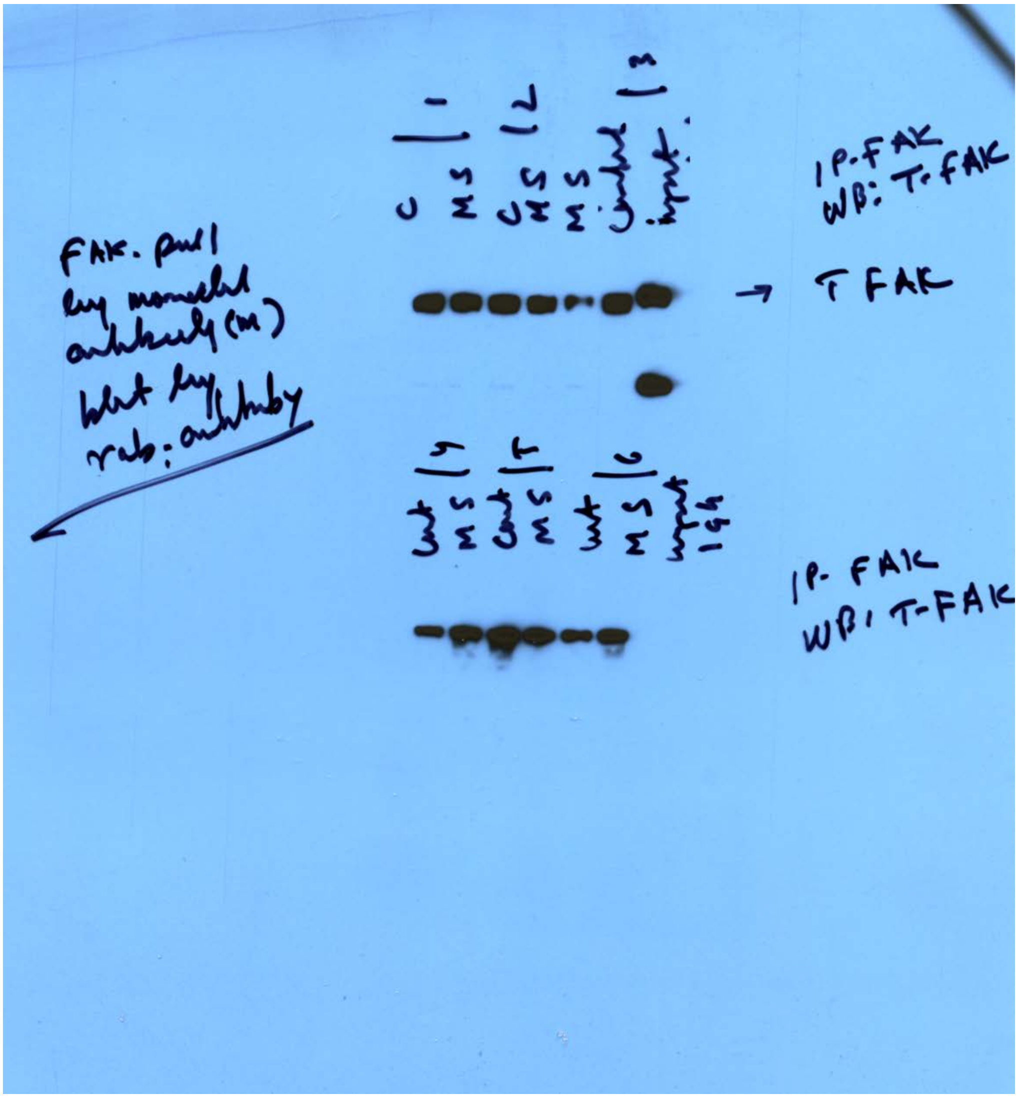

Fig. 4B

IP: ASK1 **WB: p-ASK1**

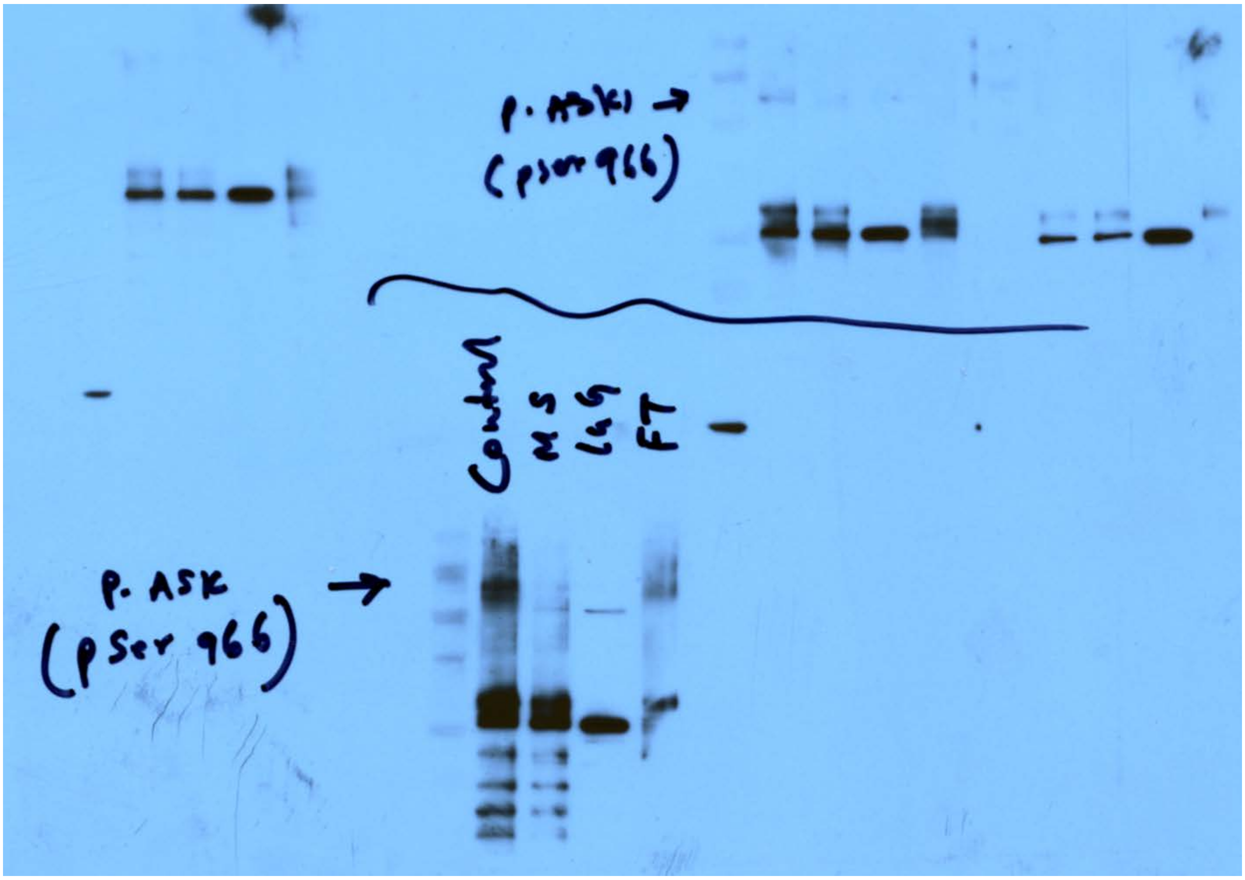

Fig. 4B **IP: ASK1** **WB: ASK1**

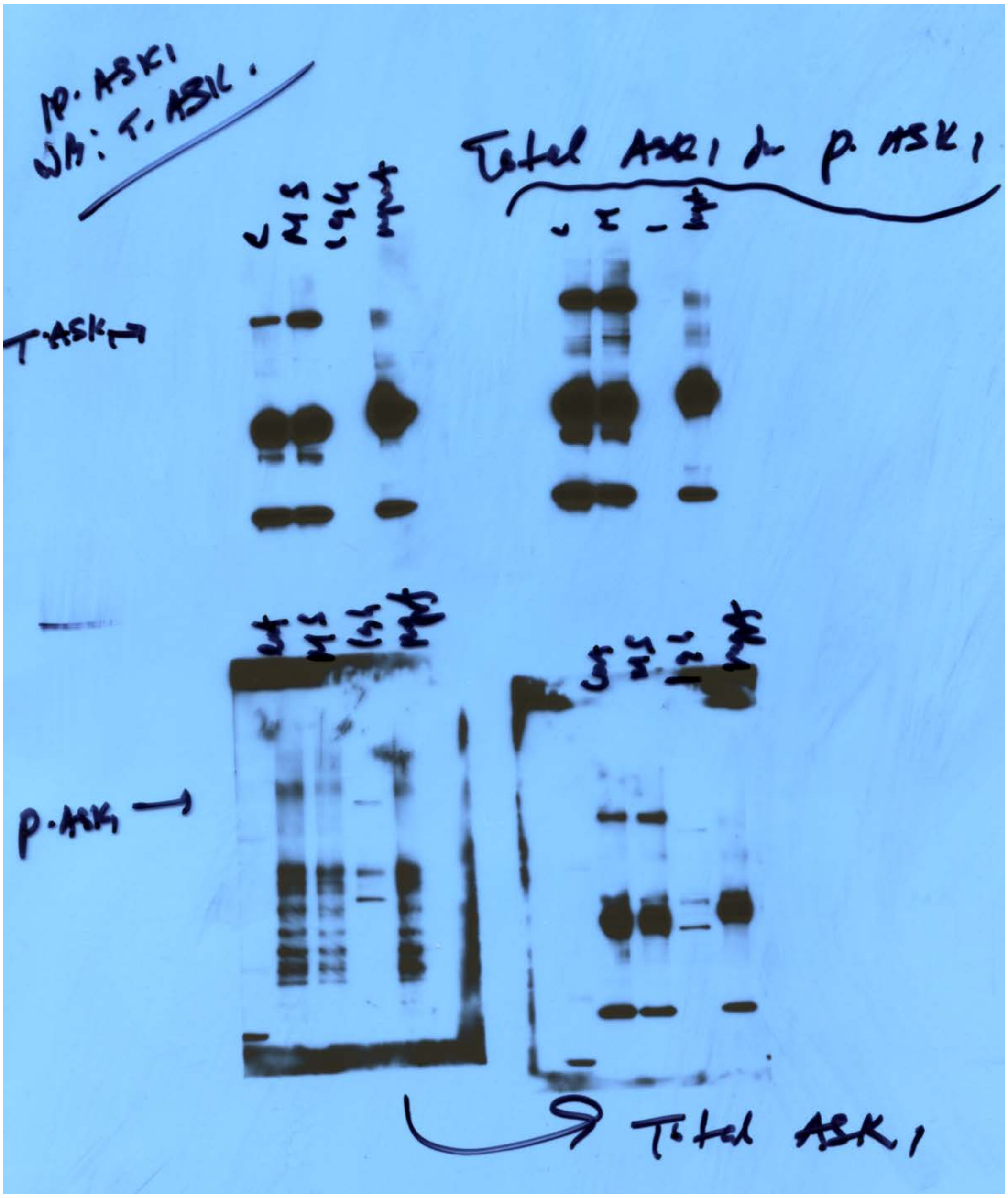

Fig. 4C **IP: FAK** **WB: p-ASK1**

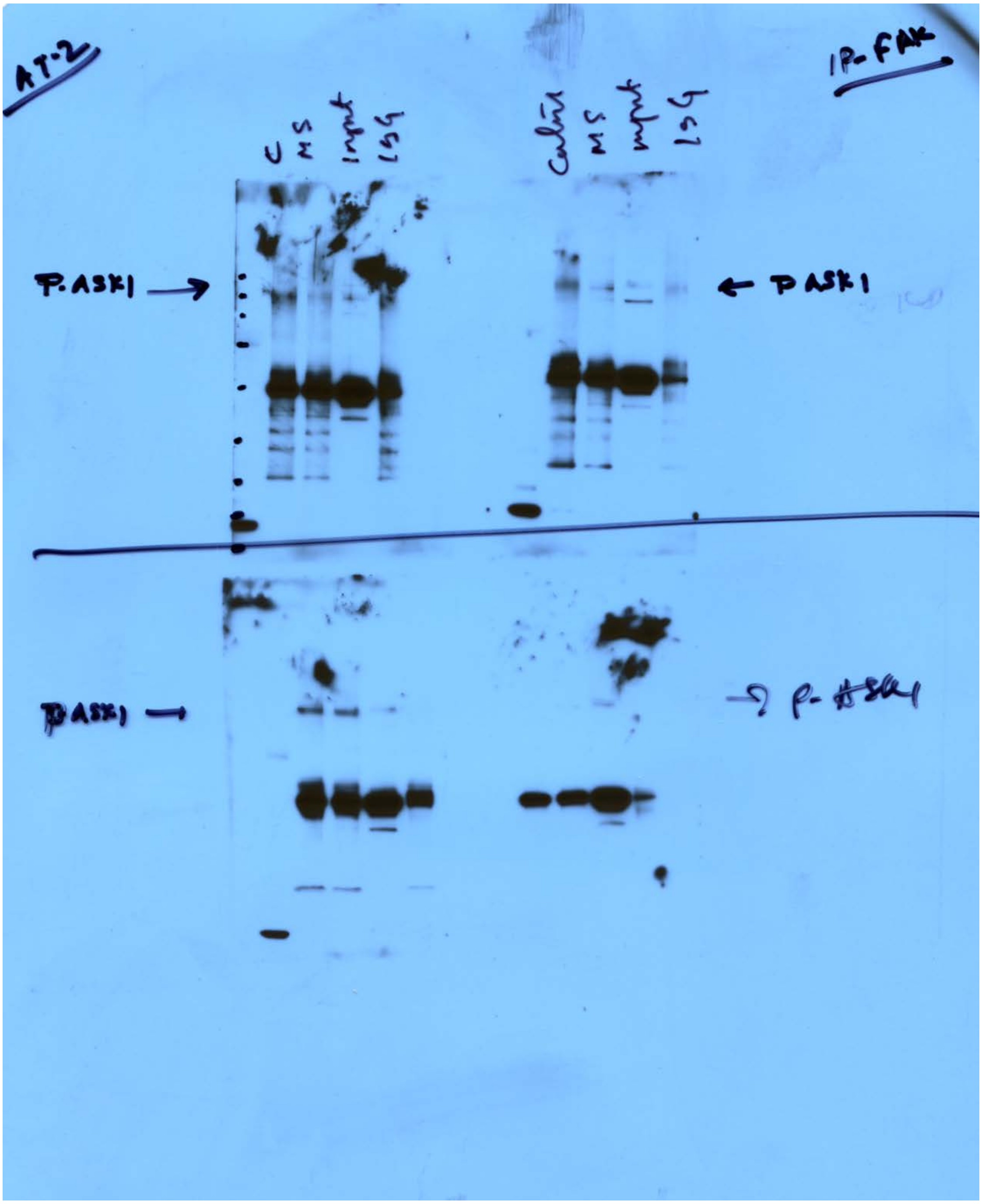

Fig. 4C **IP: FAK WB: FAK**

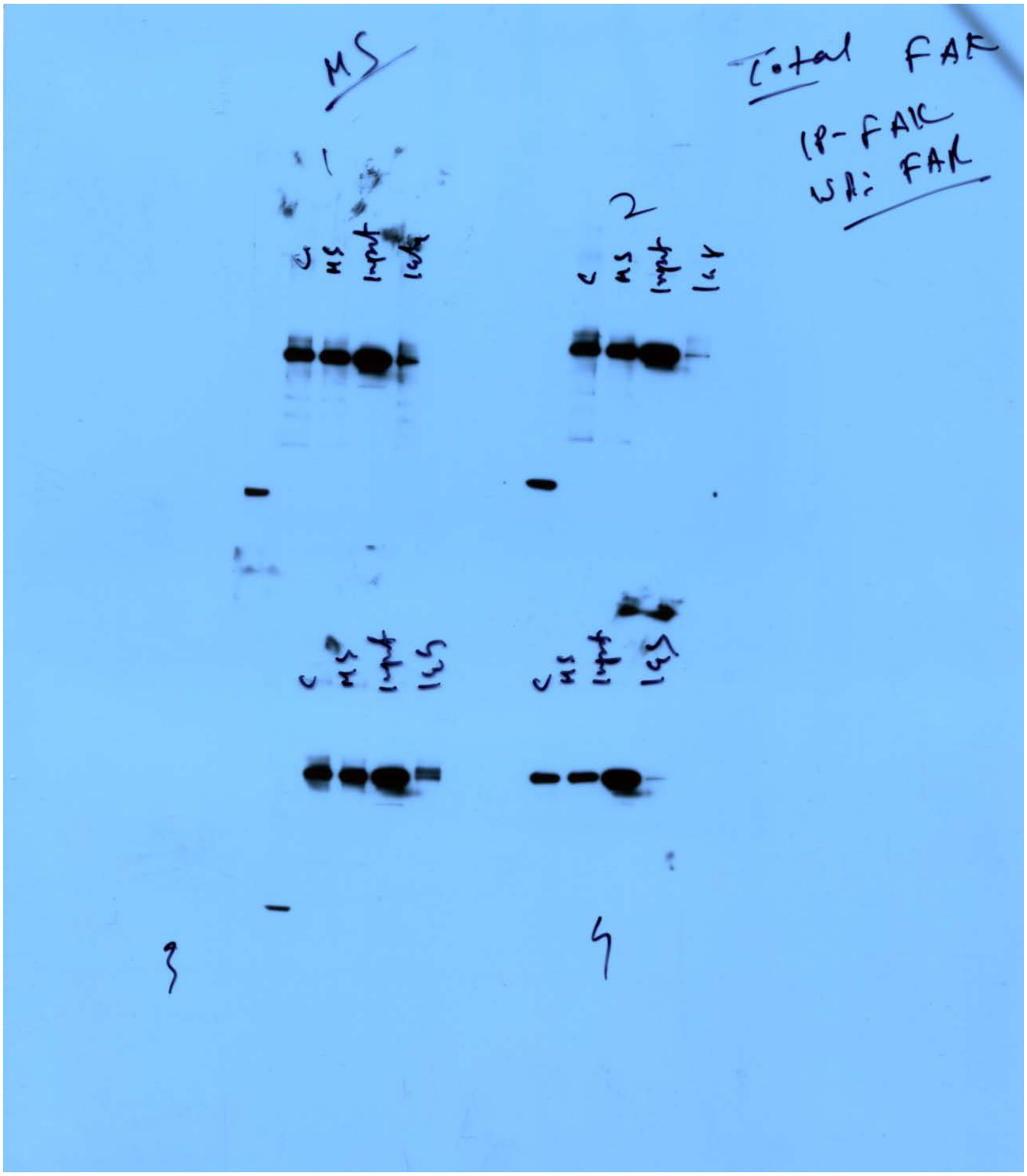

Fig. 5A

<u>IP: ASK1</u> WB: p-ASK1

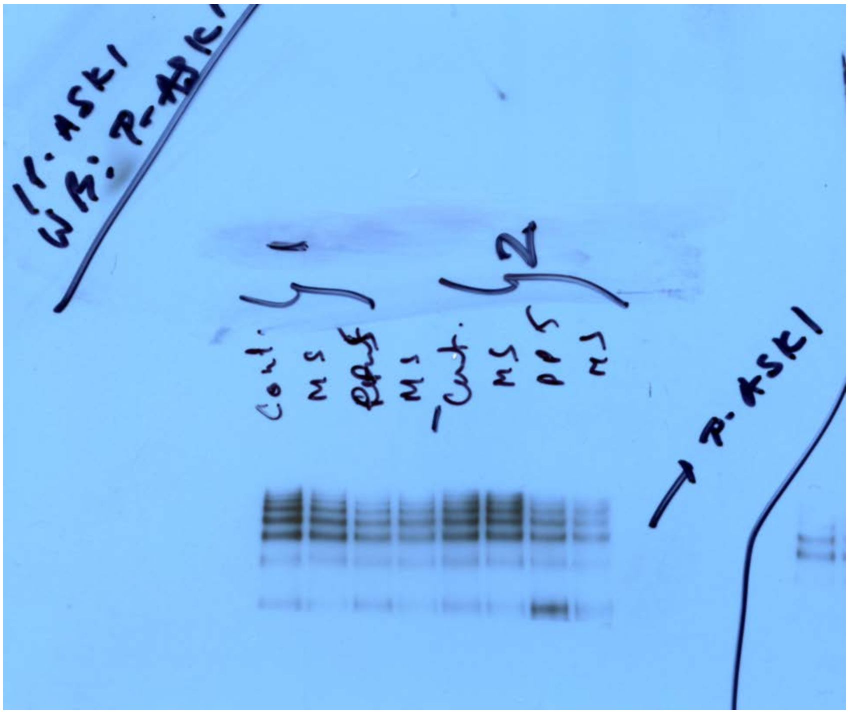

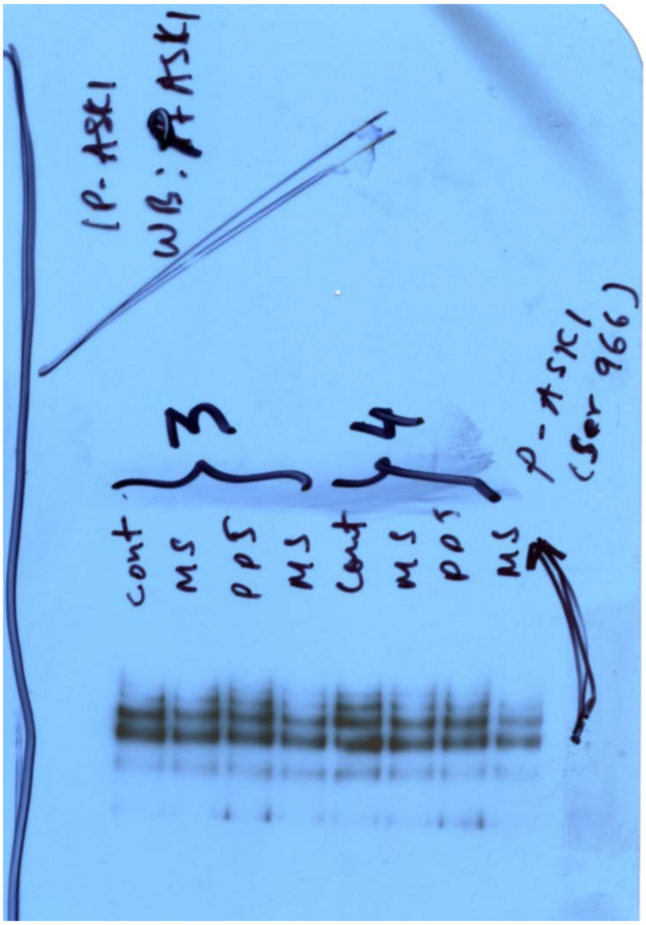

Fig. 5A **<u>IP: ASK1</u> WB: ASK1**

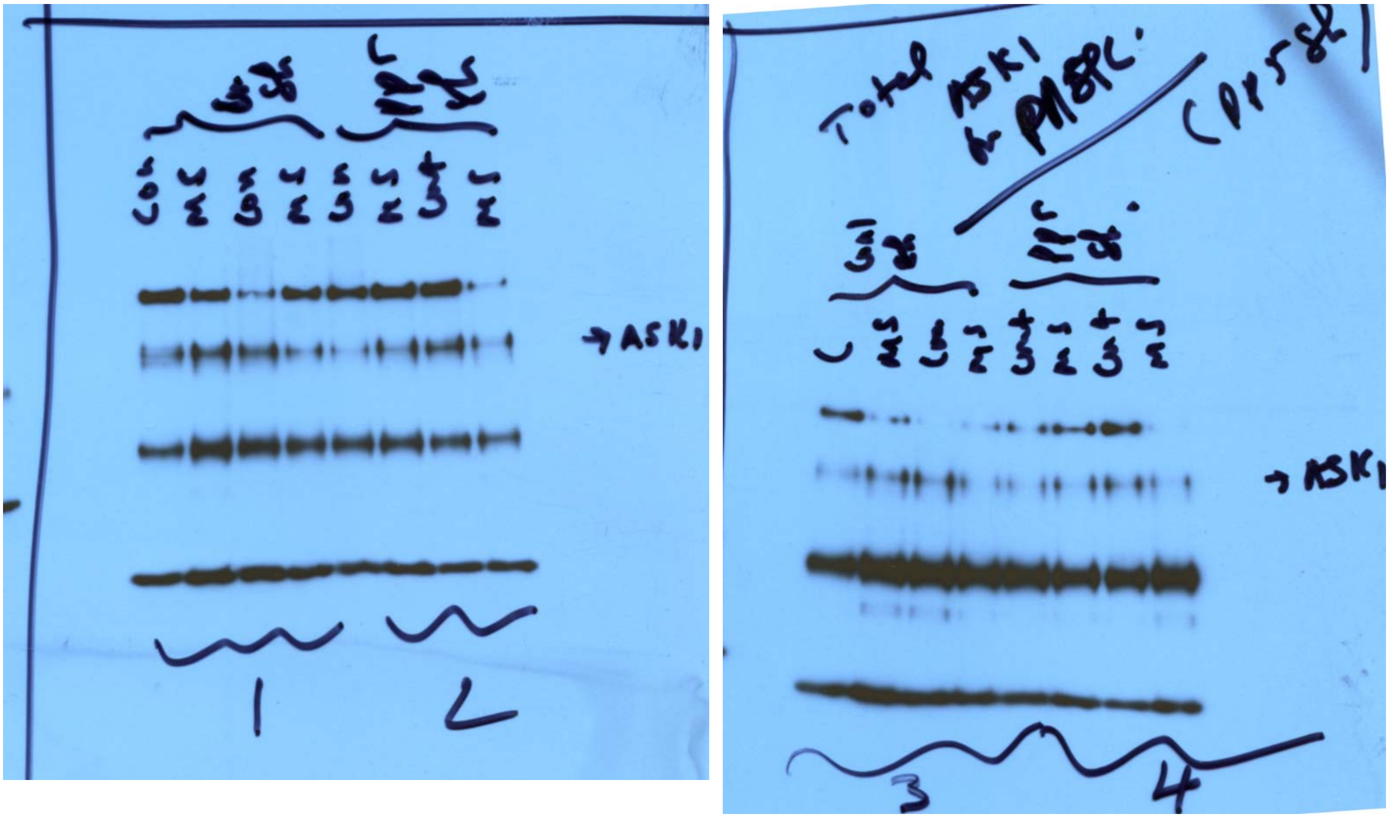

Fig. 5B **IP: FAK** **WB: p-Ser (1C-8)**

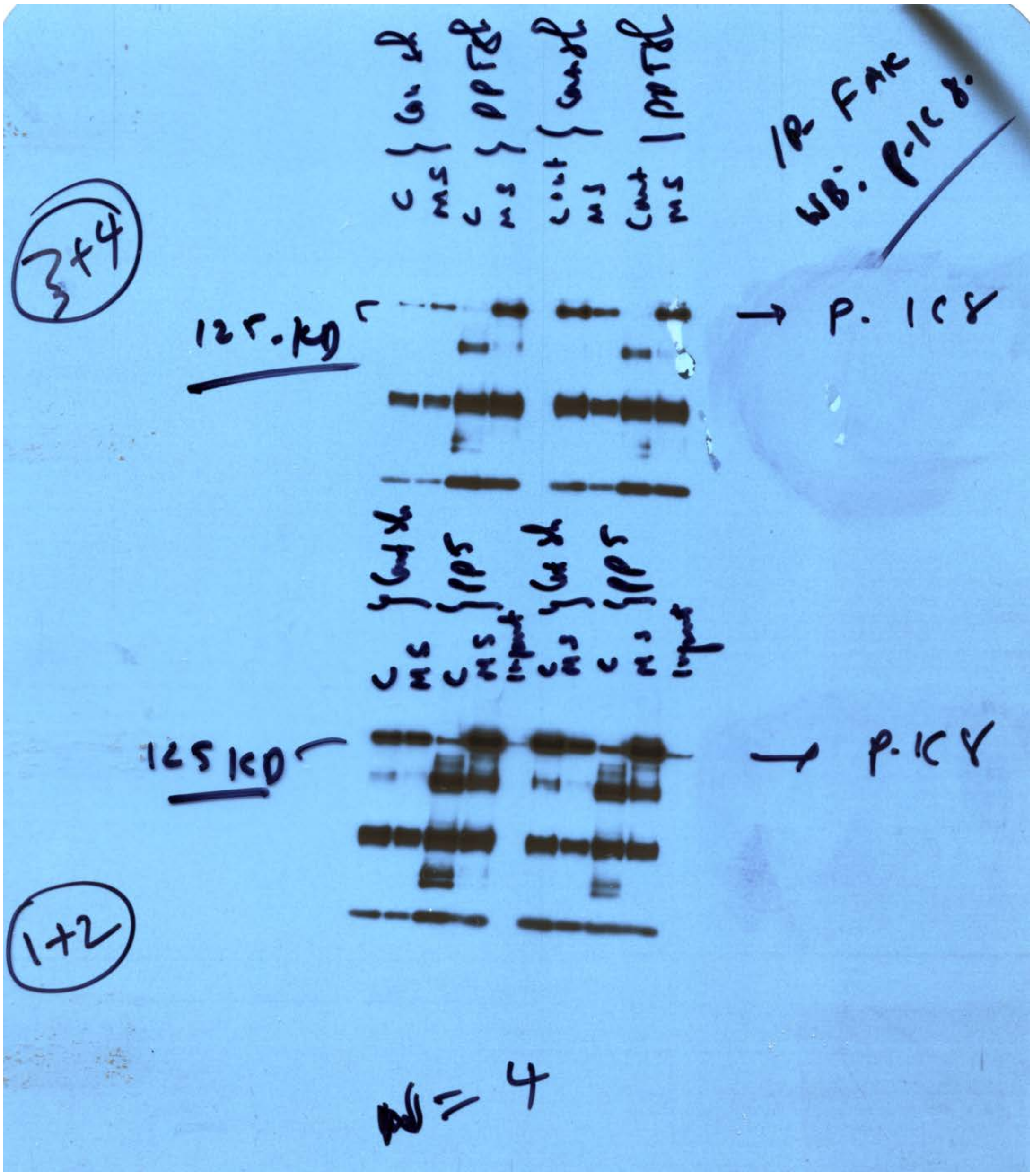

Fig. 5B **<u>IP: FAK</u> WB: FAK**

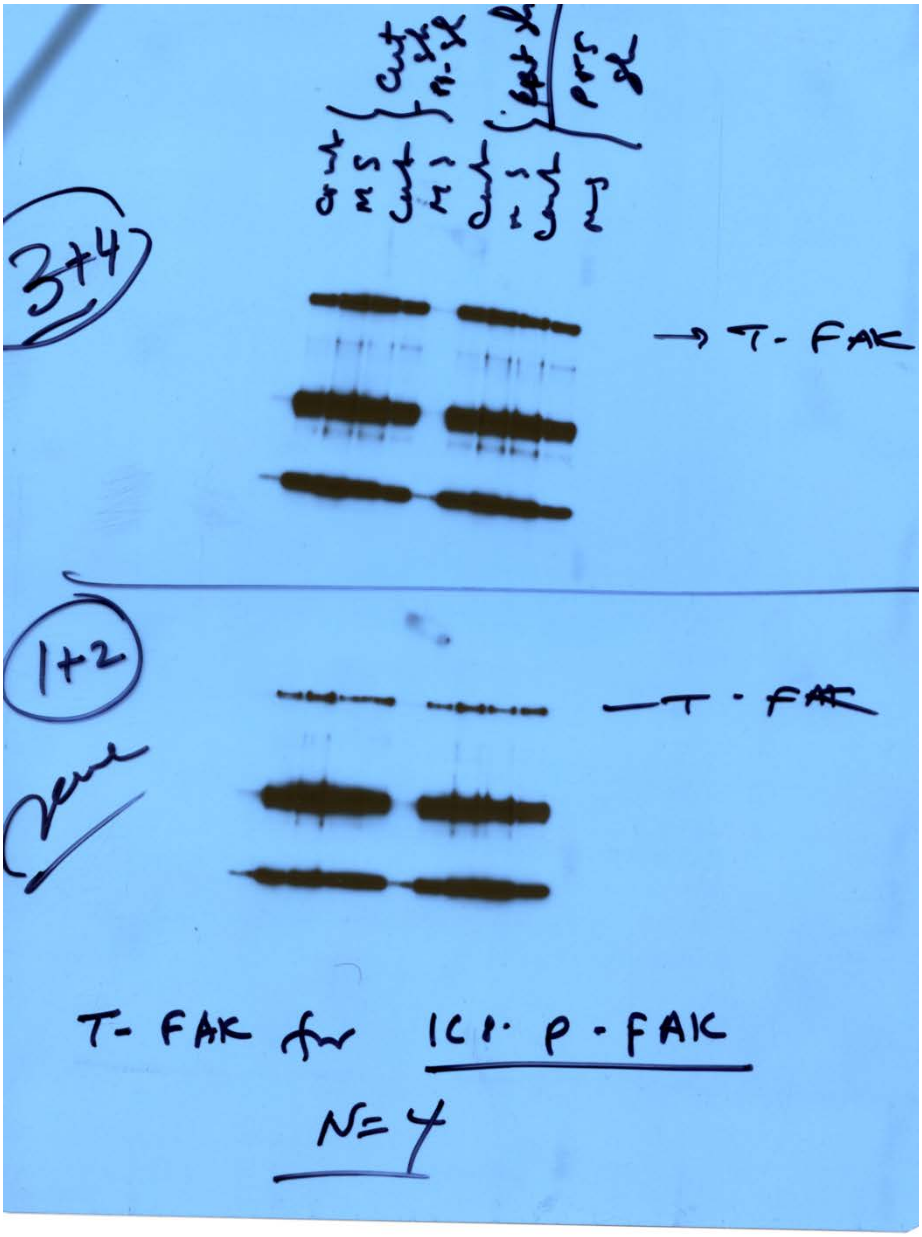

Supplement: Supplementary file 1 — Supplemental figures R1 [file 41598_2017_2204_MOESM1_ESM.pdf]
